# Analysis of Bis(trifluoromethylsulfonyl)imide Interactions with Metal Cations Through a Chemical Informatics Approach

**DOI:** 10.3390/molecules31010018

**Published:** 2025-12-20

**Authors:** Tej Gumaste, Fynn L. Cooper, James D. Blakemore

**Affiliations:** 1 Department of Chemistry, University of Kansas, 1567 Irving Hill Road, Lawrence, KS 66045, USA; 2 Department of Electrical Engineering and Computer Science, University of Kansas, 1520 West 15th Street, Lawrence, KS 66045, USA

**Keywords:** X-ray diffraction, crystallography, data science, trends, weakly coordinating anions

## Abstract

Nominally weakly coordinating anions are useful for modulating the solubility and chemical properties of metal complexes, but identification and analysis of the systematics of the interactions of anions with cationic metal complexes has not received the attention it deserves. Here, a chemical informatics approach is demonstrated for identifying and quantitatively analyzing the ways that the bis(trifluoromethylsulfonyl)imide anion (TFSI) can interact with metal-containing species. An open access computer program (PyCIFTer) was developed to facilitate large-scale structural analysis of TFSI-containing species by utilization of experimental atomic coordinate data from single-crystal X-ray diffraction (XRD) studies obtained from the Cambridge Structural Database (CSD). PyCIFTer establishes a three-dimensional vector space from the raw atomic coordinates, generating acyclic, undirected graphs that are used to rapidly analyze the structural properties (bond lengths and angles) of TFSI in individual structures in sequential/batch fashion. The structures are sorted by PyCIFTer into groups based on pre-set and chemically sensible criteria, affording a comprehensive and systematic view of TFSI structural chemistry. This approach avoids tedious one-at-a-time interrogation of structures, a prospect unreasonable in this case, and many others of contemporary chemical relevance; there were over 1500 structures in the CSD containing TFSI as of November 2024. The results demonstrate that TFSI only rarely binds to cations in the solid state, favoring the formation of species in which TFSI is found in cations’ outer coordination spheres. The prospect of applying PyCIFTer to other moieties is also discussed. PyCIFTer is also schematically compared to the commercial CSD Python application programming interface (API). Taken together, this work demonstrates the usefulness of modular workflows for sequential/batch analysis of structural data from XRD, an approach that appears poised to accelerate the translation of legacy structural results into new chemical insights and hypotheses.

## 1. Introduction

**Scheme 1:**
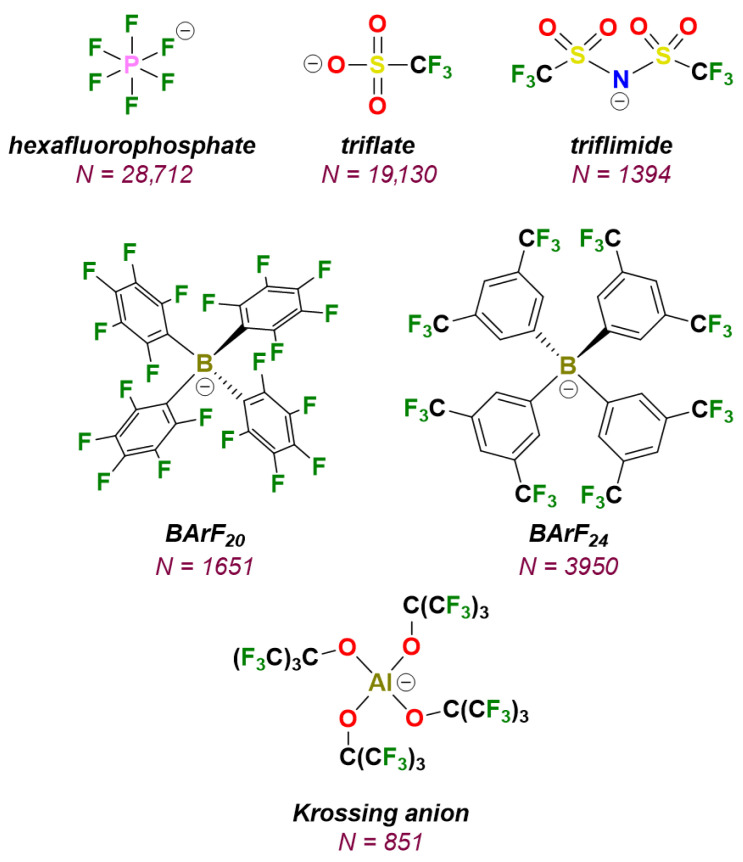
Chemical depictions of selected weakly coordinating anions (WCAs). *N* denotes the number of structures contained in the Cambridge Structural Database (CSD) that feature each anion.

Incorporation of weakly coordinating anions (WCAs) into metal-containing salts is a useful approach for tailoring chemical properties, including solubility, reactivity, and/or structural characteristics [1]. Metal salts containing WCAs are essential compounds with applications in organometallic chemistry and catalysis [2,3,4,5,6], as well as in metal-ion battery technologies [7,8,9]. Often tailormade for each specific application, a variety of WCAs have been developed; early examples include small, fluorinated anions such as BF_4_^−^, PF_6_^−^, and SbF_6_^−^ [10]. ClO_4_^−^ is another small anion that has been known to display weakly coordinating behaviors for many years as well [11,12,13]. However, more recently, bulkier anions have been developed to take advantage of the effects like size mismatch between cation and anion to induce weaker coordination. Select examples of anions of this type include trifluoromethylsulfonyl-containing variants such as triflate [14,15] (OTf^−^) and bis(trifluoromethylsulfonyl) imide [16,17,18] (TFSI^−^), as well as more exotic borane and aluminate formulations [10,19,20]. Scheme 1 shows the chemical structures of selected WCAs, including those mentioned here, along with the number of individual structures containing these chemical moieties in the Cambridge Structural Database (CSD) [21].

A common theme shared by results concerning the subset of WCAs shown in Chart 1 is the breadth of structural information obtained from crystallography that is currently available, with more than 50,000 structures from experimental work being contained in the Cambridge Structural Database (CSD) [21]. The CSD originated in a research group in 1965 within the Chemistry Department of the University of Cambridge but has grown to be an incredibly powerful resource in the intervening years [22]. The CSD is a particularly important resource for the chemistry and structural science communities, with its usefulness growing in recent years with programs like TopCryst showcasing the advanced analysis capabilities that are now available, which build on the availability of the datasets contained in the CSD [23]. In the work described here, we utilized the CSD to conduct a detailed large-scale analysis of the structures containing the TFSI^−^ motif, in that we conducted a preliminary basic search of the CSD for the TFSI anion structure and then conducted more detailed analysis in a new free-standing and open access program, PyCIFTer, using the data from the CSD as input. Our approach of developing and using PyCIFTer represents an alternative to utilization of the CSD Python application programming interface (API), a powerful and broadly useful commercial product that has been available since 2016 [24,25].

Taking advantage of such a large and diverse set of data (as found in the CSD) represents a distinctive approach towards identification and analysis of the systematics and interactions engendered by WCAs in metal-containing complexes. However, analysis of structural data of this type through, for example, chemical informatics (also known as cheminformatics) approaches has not received the attention that it deserves. In the realm of cheminformatics, a variety of extremely popular software tools have been developed to examine topics such as pharmacokinetics [26], visualization of molecular structures [27], in silico drug discovery from herbal medicines [28], deconvolution of large mass spectrometry/mass spectrometry (MS/MS) datasets [29], and materials discovery using failed experiments [30]. Each of these examples illustrates the rise of software tools for the execution of manipulations and analysis of large quantities of scientific data, particularly with regard to the correlation of molecular structural properties with apparent or achievable chemical characteristics [31]. Inspired by these recognized successes in diverse fields, we anticipated that development of a software tool capable of quickly analyzing trends in structural data from the CSD could offer an appealing strategy for gaining insights into the chemical properties of WCAs, as identified in experimental single-crystal diffraction work.

In the study reported here, we selected the bis(trifluoromethylsulfonyl)imide anion (TFSI^−^) for investigation. TFSI^−^ is an anion that we targeted for study in other experimental work, including both quantification [32] of the Lewis acidity values associated with salts of mono-, di-, and trivalent cations and synthetic investigations of the properties of uranyl complexes incorporating TFSI^−^ salts [33]. In these other works, as well as the results described here, TFSI^−^ was selected for study due to its similarity to the more commonly encountered triflate anion (OTf^−^). Triflate has been well studied from a variety of perspectives [34,35,36,37,38,39], but generally speaking, it appears that TFSI^−^ has been more rarely investigated in synthetic or structural chemistry [32]. TFSI^−^ is particularly recognized in work with ionic liquids and in battery engineering [40,41,42], but the molecular and chemical properties of TFSI^−^ species have not received the attention that they deserve.

Here, we report the development of a computer program, titled PyCIFTer, for large-scale structural analysis of TFSI-containing species on the basis of X-ray diffraction (XRD) data available in the Cambridge Structural Database (CSD). PyCIFTer establishes a three-dimensional vector space from the raw atomic coordinates available in the CSD structures and utilizes a wave-emanating search procedure to enable rapid analysis of bond lengths and angles of TFSI-containing species, as well as examination of trends in the available structural data. Structures can be sorted by PyCIFTer into groups based on pre-set and chemically sensible criteria, affording a comprehensive and systematic view of the structural chemistry of TFSI^−^. Based on the observed trends, conclusions can be drawn that support the formulation of hypotheses regarding the chemical influence that TFSI^−^ may have on chemical systems, and several of these are discussed here. In this paper, we explain our development of PyCIFTer (Section 2.1, Section 2.2, Section 2.3, Section 2.4, Section 2.5, Section 2.6 and Section 2.7), show results obtained with PyCIFTer regarding the TFSI^−^ motif and related WCAs (Section 2.8, Section 2.9 and Section 2.10, compare features of PyCIFTer to those of the CSD Python API (Section 2.11) [24,25], and provide an outlook on future work (Section 2.12). Taken together, the results show that a chemical informatics approach can avoid the need for tedious one-at-a-time interrogation of XRD structures from the CSD, suggesting opportunities for the development of open access data science tools that accelerate structurally motivated research into the properties of particular chemical species, like weakly coordinating anions (WCAs).

## 2. Results and Discussion

### 2.1. Qualitative View of the Structural Diversity of the TFSI Anion

**Scheme 2:**
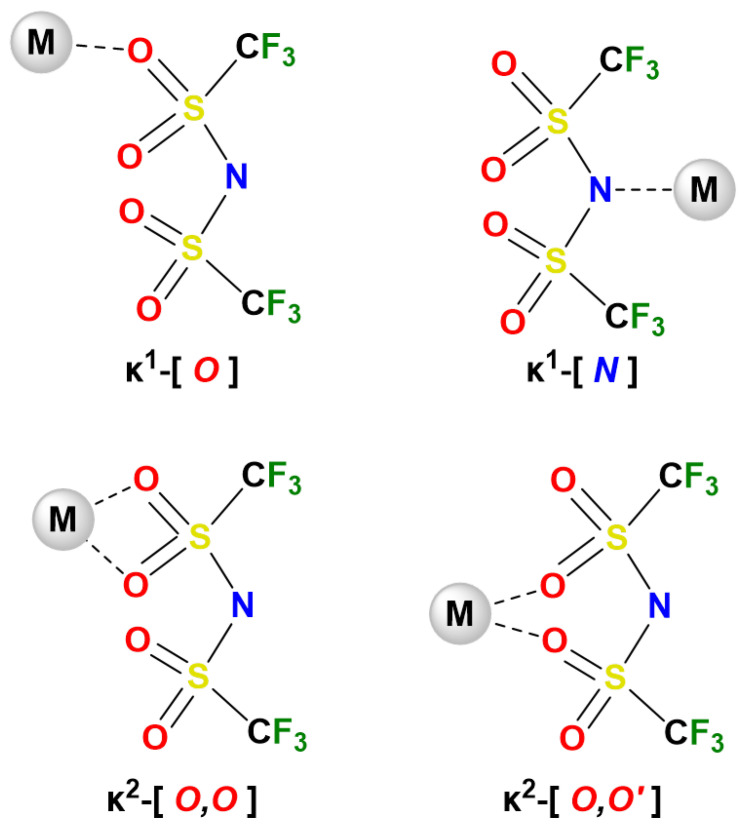
Coordination modes in which TFSI^−^ can participate with a general metal center.

From the perspective of structural diversity, a qualitative inspection of structures in the CSD containing TFSI^−^ shows that there are at least four coordination modes that this anion can adopt when paired with metal cations. These possible coordination modes are shown schematically in Scheme 2. We note, however, that even our qualitative review of TFSI-containing structures in the CSD revealed many examples in which the TFSI core motif was not bound to a paired cation, but rather found in the outer coordination sphere, in accord with its tendency to serve as a WCA. Nonetheless, TFSI^−^ can be found in the first coordination sphere of cations. Two of the possible coordination modes in this respect show denticities of κ^1^, wherein TFSI^−^ interacts directly with metal cations through either a single oxygen atom (as in the κ^1^-[O] mode) contained in one of the trifluoromethylsulfonyl moieties or the single nitrogen atom contained in TFSI^−^ (as in the κ^1^-[N] mode). There are also at least two conceivable bidentate coordination modes, wherein TFSI^−^ interacts with two oxygen atoms on the same trifluoromethylsulfonyl moiety (as in the κ^2^-[O,O] mode) or with two oxygen atoms on different trifluoromethylsulfonyl moieties contained in the same TFSI^−^ species (as in the κ^2^-[O,O′] mode). For clarity, we have included figures illustrating example structures of several of these possible coordination modes here (vide infra).

**Figure 1:**
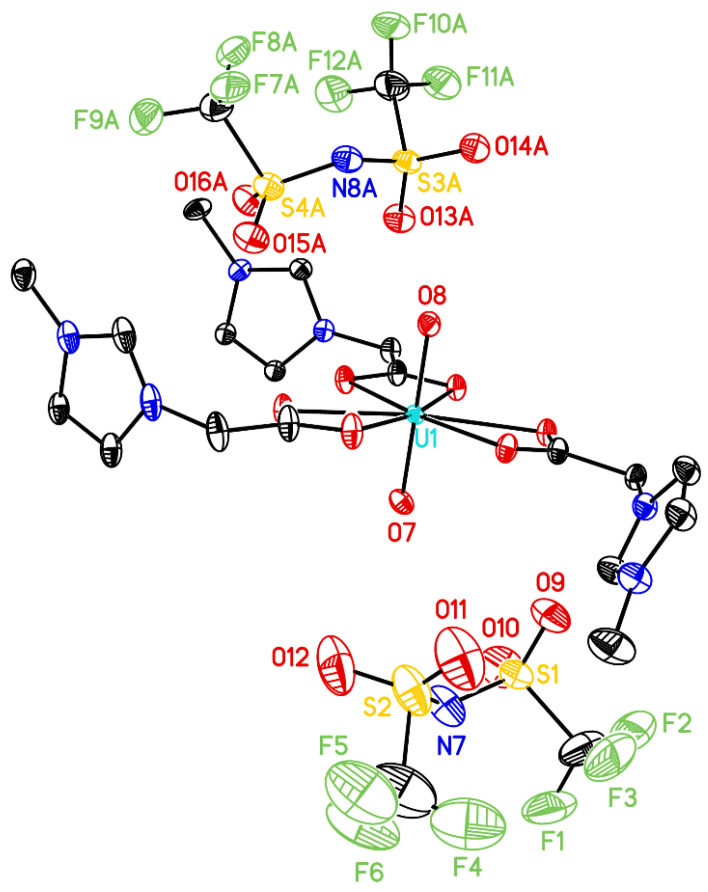
Solid-state structure from XRD analysis in which two TFSI anions are found in the secondary coordination sphere of a formally dicationic uranyl complex. All hydrogen atoms are omitted for clarity, and displacement ellipsoids are shown at the 20% probability level. From reference [43,44].

**Figure 2:**
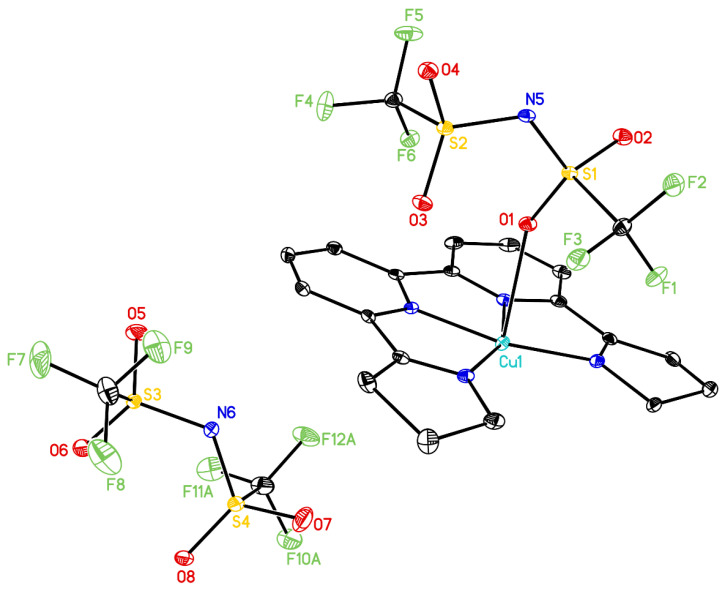
Solid-state structure from XRD analysis in which one TFSI anion is coordinated to a copper complex in the κ^1^-[O] mode (through oxygen atom O1). A second TFSI anion is found in the outer coordination sphere. All hydrogen atoms are omitted for clarity, and displacement ellipsoids are shown at the 20% probability level. From reference [45].

**Figure 3:**
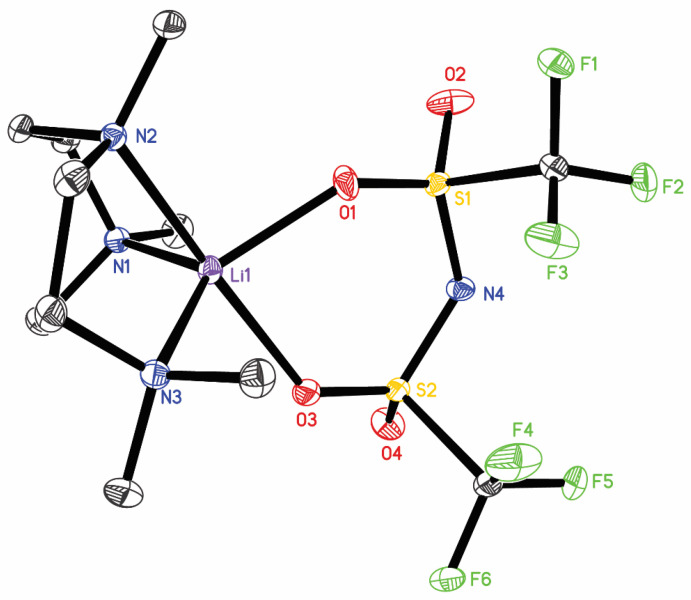
Solid-state structure from XRD analysis in which one TFSI anion is coordinated to a lithium complex in the κ^2^-[O,O′] mode (through oxygen atoms O1 and O3). All hydrogen atoms are omitted for clarity, and displacement ellipsoids are shown at the 20% probability level. From reference [46].

Figure 1 shows an example structure in which TFSI^−^ is found in the outer coordination sphere of a uranyl (UO_2_^2+^) compound [43,44]. In this species, the first coordination sphere of the uranyl ion is fully occupied by three carboxylate donors, as well as the two conventional oxo moieties held in a trans configuration. In this structure, the uranyl-containing species holds an overall charge of 2+ due to the presence of three methylated imidazole rings on the periphery of the complex, and thus the two TFSI^−^ moieties support charge balance in the final structure. Figure 2 shows an example in which one TFSI^−^ moiety is found in the outer coordination sphere of a Cu(II) complex, but in this case, the second equivalent of TFSI^−^ associated with this compound is bound to the copper center in the κ^1^-[O] mode [45]. This structure illustrates that TFSI^−^ can be found in different environments in the same compound, and that the coordination mode adopted by the different equivalents need not be the same to result in an isolable compound. And, as the final example shared here, Figure 3 shows an example in which one TFSI^−^ moiety is found in the inner coordination sphere of a lithium complex, and is coordinated to the lithium center via the κ^2^-[O,O′] mode [46]. As these example structures demonstrate, the TFSI^−^ moiety can be reasonably viewed as promiscuous in its coordination chemistry—readily able to bind to cations in an inner-sphere fashion and through a variety of coordination modes. And, of course, we emphasize that the structure shown in Figure 2 demonstrates that the TFSI^−^ moiety can prefer to be localized in the outer coordination sphere of cationic metal complexes, even those like the copper complex in Figure 2, in which there appear to be possible open coordination sites at which TFSI^−^ might have been otherwise predicted to bind in an inner-sphere fashion. Considering all this, a primary motivation for our project was to ascertain, through a chemical informatics approach, if it can be reliably concluded that the TFSI anion prefers to be found in the outer coordination spheres of cationic species, a result that would be in accordance with certain experimental titration results from our laboratory [32]. Certainly, we were interested in ascertaining whether a comprehensive view of the available structural data from single-crystal X-ray diffraction analysis would confirm whether this might be the case, at least in the solid state.

### 2.2. Source Data from the Cambridge Structural Database

The technical work of the project described here was carried out using data contained in a set of data files which were of the standardized Crystallographic Information File (CIF) type. CIF-format files are commonly encountered in inorganic and organometallic chemistry (and in other fields), as their format is the standard for reporting structural data from X-ray diffraction (XRD) analysis. Virtually all modern solid-state structures from XRD analysis are contained in the Cambridge Structural Database (CSD), making it an extremely convenient and useful source of data for use in chemistry, materials science, and crystal engineering [21]. The data science details of CIF-format files have been extensively documented elsewhere [47,48], but as this paper has been written from an interdisciplinary perspective meant to bridge chemistry and computer science, we begin by describing some of the key aspects of the contents of this file type from a chemical perspective.

The first 35–37 lines of code in the CIF format contains bibliographical and nomenclature information about the structure described in the file. This first section of the file is variable in terms of the total number of lines of code due to the need to report certain structural details for some compounds, but not all. Key elements found in this header for all compounds include the name of the primary compound featured in the structure, as well as bibliographic information associated with the publication in which the structure is reported (including author names, journal name, first page of the article, etc.). The next 20 lines contain general physical information about the crystal that was diffracted in the experiment, leading to the present CIF-format file. These data include details on the crystallographic unit cell [49] that are not strictly relevant to this project, including cell volume, experimental crystal color, experimental crystal density, and other parameters.

After the previously noted section pertaining to general physical information, one comes to the critical section of the CIF-format file for this project. These 15–20 lines contain the dimensions of the unit cell, which are integral to the function of PyCIFTer; the unit cell will be used to calculate bond distances and angles by the program (vide infra). In this section, atomic radius values are included as well. These radius values can be used to estimate whether elements found in the solid-state structure are bonded to each other [50]. PyCIFTer does not rely on the default radii provided in the CIF-format files from the CSD, but instead uses element-specific covalent radii from a comprehensive review of recommended values of covalent radii from 2008 [51]. We elected to utilize the values from the 2008 report that are available in the noted peer-reviewed paper for two reasons. First, consideration and selection of appropriate values for the covalent radii gave a valuable opportunity to closely look at how select TFSI moieties interact with their surroundings in the solid state. Second, reviewing the comprehensive list of radius values and compiling the relevant values for our work facilitated development of the code for PyCIFTer, as values for all relevant elements on the periodic table were loaded into the program prior to execution of the supervised analyses.

Finally, the last section of the CIF-format file consists of rows of five space-separated values, each of which corresponds to a specific property for each atom in the compound in the structure. In this section, PyCIFTer utilizes the (x, y, z) coordinates for each atom in the structure, as well as labeling that tells the program the identity of the element at each position. The (x, y, z) coordinates in this case are fractional spherical coordinates, and conversion of these into Cartesian coordinates, as well as atom-to-atom distances, were crucial steps in our project that are described below in some detail.

### 2.3. Summary of Relevant File Syntax in the CIF Format

The first task of the data science dimension of this project was to create the algorithms needed for parsing the information contained within the file structure of the CIF format. Each CIF was obtained from ConQuest [52], a highly refined and capable structure querying program marketed by the Cambridge Crystallographic Data Centre to visualize structures in three-dimensional formats. As described above, CIFs include bibliographic, chemical, crystal, experimental, refinement, and atomic coordinate data resulting from the CSD’s validation procedures and data processing.

**Figure 4:**
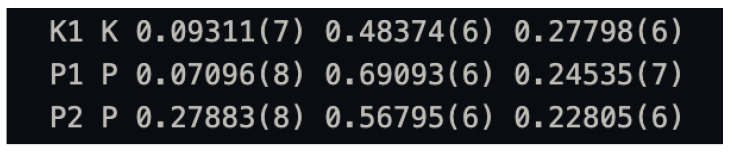
Sample spherical (x, y, z) coordinates for three atoms in Zone I of the CIF corresponding to CSD structure ACIHIE from reference [53,54]. The first entry is the atom label, the second entry is the element identity (scattering factor), and the three numerical entries are the (x, y, z) spherical coordinates with their estimated standard deviations (ESDs).

This project utilized two major regions of the CIF. The first region contained a text-based grid of symbols and numbers, which is designated as Zone I for the purposes of this discussion (see Figure 4). Each row corresponds to a given atom and its properties, as shown in Figure 1. The columns are interpreted as follows: label, symbol, X-coordinate, Y-coordinate, Z-coordinate. The coordinate system used in the file is spherical (vide infra). For the purposes of the program described here, the label associated with an atom is denoted as an “identifier,” a term synonymous with chemists’ routine referencing of “atom names.” The identifier consists of the atom symbol and an index which increases with multiple instances of the same element in a molecule—for example, C1, C2, etc.

**Figure 5:**
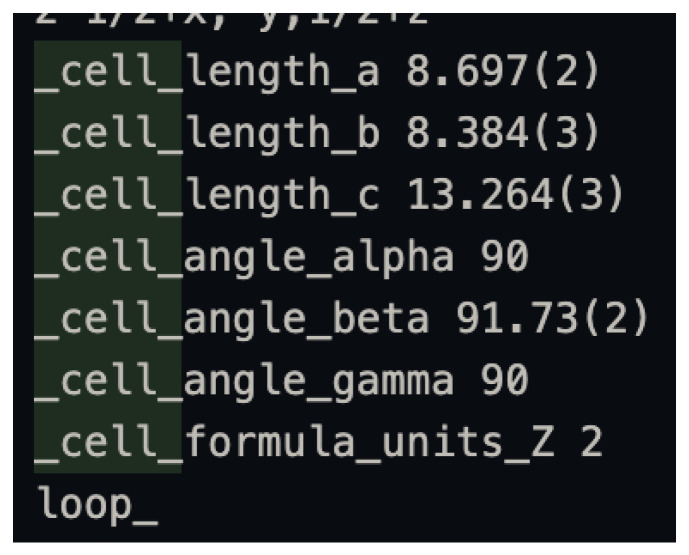
Sample unit cell parameters and values of the parameters from Zone II in the CIF corresponding to structure ACIHIE from reference [53,54].

The second region, designated as Zone II for this discussion, lies above the grid of individual atom information and consists of seven lines containing the key parameters describing the unit cell of the crystal containing the molecule(s) of interest in a given structure (see Figure 5). The unit cell parameters of the molecule are used in PyCIFTer, as in other contexts in the field of chemical crystallography, to retrieve the Cartesian coordinates of each atom. In turn, of course, these Cartesian coordinates can be used to determine bond distances, interatomic spacing, angles, etc. The lengths of the unit cell are given in Angstroms (Å; 10^−10^ m), and the unit cell angles are given in degrees (°).

### 2.4. CIF-Format Data: Source and Operations

The ca. 650 CIF-format files used as the source material for input into PyCIFTer and the analysis presented here were obtained from a structure-based search of the CSD using ConQuest. The atom-to-atom connectivity of the TFSI anion was input into the structure query tool, using the appropriate single- and double-bond notations for each connection. Importantly, in order to simplify the interpretation of results from PyCIFTer, all structures involving disorder in the solid state (as flagged within the CSD) were removed from consideration in the ConQuest search. Although this choice sacrifices some data from the CSD and narrows the interpretation of our study, it was decided that this approach avoids certain difficulties that would have impeded basic development of PyCIFTer. These include the challenge of interpreting atom-to-atom connectivity in congested structural data in which, for example, multiple isomers of the same compound or otherwise related species are co-crystallized in the same volume of the crystallographic asymmetric unit.

Another simplification that should probably be mentioned concerns the interpretation of the space group symmetry of the solid-state structures used for the analysis carried out by PyCIFTer. PyCIFTer was written to only interpret the atoms explicitly included in the asymmetric unit(s) described in the CIF-format data obtained from the CSD. In particular, PyCIFTer examines the data for S–N–S moieties that are associated with TFSI species. PyCIFTer does not extrapolate unit cell contents and thus considers only those atoms that are explicitly defined in the atom list contained in a given CIF-format file. This approach avoids the complexity of applying symmetry operations to the asymmetric unit contents found in the files. Along this line, it should be mentioned that, if a structure were to feature a TFSI moiety (containing an individual S–N–S unit) on a position featuring a mirror plane or *C*_2_ rotation, for example, PyCIFTer would not flag that moiety as being associated with a TFSI species for analysis. This is because PyCIFTer requires the explicit definition of all three atoms in a given S–N–S moiety. If TFSI were to be found in the data at a position involving such a symmetry element, only the coordinates of the atoms associated with one [SO_2_CF_3_] group would be defined in the solid-state data, along with the atomic coordinates for the central [N] of the TFSI. The central N would, of course, have an occupancy factor of 0.5 as well. PyCIFTer does not apply space group symmetry to the asymmetric unit to widen its search for S–N–S moieties, meaning that any TFSI species located on a crystallographic symmetry element in this way is not flagged for further consideration. A future version of PyCIFTer could be developed to include consideration of space group symmetry, but our goal in this work was to focus on basic functionality relevant to consideration of the coordination modes of TFSI.

As mentioned above, the geometric data in the CIF that describes the relative positions of the atoms in the structure are presented in the spherical coordinate system. The (x, y, z) coordinates of each atom represent their positions as individual points in the spherical system. To retrieve the Cartesian coordinates of each atom in a CIF, the CIFParser class of PyCIFTer establishes an invertible matrix transformation function whose inputs are the cell parameters of the unit cell and a special quantity called α*. The matrix transformation (CM) and the expression for α* is denoted below in Equations (1) and (3) [55].

Each CIF’s path is passed to the CIFParser class in cifFileParser.py. Each molecule is represented by an instance of this class [56]. The class offers utility functions that allow for easier manipulation of the atomic position data and serve as an abstraction for each molecule. Here, abstraction refers to the process of taking into consideration only the relevant details of a given entity, in this case, the molecule(s) of interest. For example, in the analysis presented here, only molecular geometries are relevant, not details on crystal quality or the refinement model used to obtain the structure reported to the CSD. The CIF is split into two different parts based on the index—say, start index—of the row containing “_atom_site_fract_z” keyword. All lines before this one contain the metadata—surrounding properties of the CIF like the cell values—of the CIF, while the lines after the start index contain the positional data of the structure. PyCIFTer utilizes this specific keyword to validate the presence of positional data in each CIF. The program loops through the lines before the start index, to search for lines beginning with “_cell_values_” and create a HashMap to store the cell values of the unit cell. A HashMap [57] is a data structure used for quick data access and is a collection of key–value pairs [58]. Generally speaking, HashMaps are useful for storing large amounts of labeled data as the label for the data can be stored as the key and the data itself can be stored as a value in a key-value entry of a given HashMap. In our case, individual unit-cell values are stored according to the following schema, where the curly braces indicate the usage of a HashMap.

$$\left\{CellValueName:\;<numericavalue>\right\}$$

Once the cell values are extracted, PyCIFTer builds the conversion matrix, denoted CM and referenced below in Equation (2), with all the cell values from the HashMap built previously. To prevent repetitive syntax and uphold good software engineering practices, the CIFParser class offers helper/accessor functions for some terms of the conversion matrix. An accessor function is a special type of function that is used to perform one-way access of a data member/variable in a class. Specifically, accessor functions only allow the program to retrieve a particular data value.

(1) $$CM=\left[\begin{array}{ccc} a & b\cos\gamma & c\cos\beta \\ 0 & b\sin\gamma & -c\sin\beta \cos\alpha *\\ 0 & 0 & c\sin\beta \sin\alpha * \end{array}\right]$$

(2) $$\left[\begin{array}{c} C_{x}\\ C_{y}\\ C_{z} \end{array}\right]=CM\cdot \;\left[\begin{array}{c} S_{x}\\ S_{y}\\ S_{z} \end{array}\right]$$

(3) $$\alpha *=\frac{\cos\beta \cdot \cos\gamma -\cos\alpha }{\sin\beta \;\cdot \sin\gamma }$$

The conversion matrix (*CM*) transformation used to convert the spherical coordinates to Cartesian coordinates is shown in Equation (1). Equation (2) denotes the terms *S_x_*/*S_y_*/*S_z_* for the spherical coordinates of each atom as provided in the CIF, and *C_x_*/*C_y_*/*C_z_* denote the corresponding Cartesian coordinates retrieved after applying the *CM* matrix transformation. The variable α* represents a simplification of the mathematical operations in *CM* performed to accomplish the conversion from spherical to Cartesian coordinates (Equation (3)).

At this point in the programmed workflow, the atoms are now ready to be processed from spherical coordinates to the needed Cartesian coordinate system. In this case, the conversion matrix is initialized as a NumPy [59] matrix to allow for smooth matrix operations. The program splits the file into two halves around the start index. Furthermore, each row is split based on the spaces between them to prepare the atomic information to be passed to the Atom class. The conversion matrix (CM) is passed down to the Atom constructor, which will be used to convert the extracted coordinates to Cartesian coordinates. Instead of building the conversion matrix for each atom, it is built by design in the CIFParser class, as the unit cell parameters in the conversion matrix are properties of the overall molecule and not each atom. Additionally, this ensures that the conversion matrix (CM) built for a molecule is used for all its atoms; this prevents redundant matrix operations and ensures uniform data processing with the correct CM for a given structure/CIF. The atom objects are now ready to be built using the Atom class’s constructor. (A constructor is a special characteristic function belonging to each class that initializes an instance of the class with values supplied to the constructor.)

### 2.5. Defining the Abstractions for Each Atom in the Structures

The Atom constructor receives the following: a list with an individual data row containing values that are split or separated by the space character, the conversion matrix, the metal covalent radius HashMap, and the non-metal covalent radii HashMap. The **covalentRadii** HashMap contains the covalent radii for individual metals expressed in Å [51]. Below is the schema for both the HashMaps:

$$\left\{Element\;Symbol:\;<Radius\;Value\;in\;Å>\right\}$$

The Atom class serves as an encapsulation of each atom’s properties while also providing a set of several utility methods for atom manipulation. Each atom generated is added to a **self.Atoms** master list, unique to each CIFParser class, to ensure that the reference to these atoms does not vanish after memory allocation—meaning that the atoms being processed are held in computer memory for use in further operational steps requested by the program. The CIFParser class also contains the following auxiliary methods for effective atomic manipulation:

- getElementAtoms(self, symbol): returns a list of atoms with a particular symbol after linearly searching through **self.Atoms[]**.
- containsAtom(self, symbol): checks whether the current molecule contains an atom of given symbol.
- getAtomsInARadius(self, targetAtom, radius): returns a list of atoms after performing a wave emanating search of size **radius**. The function looks for all atoms with a Euclidean distance of less than **radius** and returns a list of atoms satisfying this condition. The Euclidean distance between two points $x_{1},\;y_{1}$ and $x_{2},\;y_{2}$ is defined by the following equation:

(4) $$\sqrt{\left\{{\left.\left(x_{1}-x_{2}\right.\right)}^{2}+{\left.\left(y_{1}-y_{2}\right.\right)}^{2}\right\}}$$

- getParticularAtom(self, identifier): returns a particular atom object based on the **identifier** provided to the function (ex. C57).

The Atom class serves as an abstraction for each atom of a molecule and itself is represented by multiple class variables and methods. In the Atom constructor, the previously mentioned inputs are used to extract each atom’s instance variables—variables that are characteristic to each atom—and most importantly, it generates the position vector for the atom. The first term of a row from Zone I is subjected to word manipulation techniques to reliably extract the atom’s identifier, symbol, and atomic index—which is the index of a given element’s atom; for example, in C57, the index would be 57. After extracting the identifier, the CM matrix transformation is applied to the spherical positional coordinates to obtain the Cartesian positional coordinates of the atom. Each coordinate is rounded to six decimal places to reduce floating point errors and reduce storage inefficiencies. Lastly, the atom is assigned its appropriate covalent radius value based on its presence in the **covalentRadii** HashMap. If the atom corresponds to an element that does not exist in the HashMap, then it is assigned a radius of 0, as any elements near the nitrogen in the [S–N–S] moiety of the TFSI anion that are not metals are not of concern here. This is because the current project is focusing on the tendency of TFSI to bind to metal cations, and thus all metals that were found in at least one structure in the entire dataset pulled from the CSD (vide supra) for this analysis were pre-loaded into the **covalentRadii** HashMap.

The Atom class was built with a few utilities of its own to facilitate atom-based operations (mainly hashing and equality) more effectively. Hashing is the process of passing a variable-length input argument into a function to generate a fixed-length output. In the context of PyCIFTer, hashing is used to differentiate between each atom and is performed by using the atoms’ individual position vectors. For a given structure, no two atoms can have the exact same positional vector, eliminating any possibility of hashing collisions [36]. A subset of the utility functions is listed below:

- getDistance(self, other): returns the Euclidean distance between the position vectors of the current atom and the **other** atom.
- __eq__(self, other): operator overload for the “==” operator. This operator makes it so that if two atoms are compared using the “==” operator, the output depends on whether the two atoms have the exact same position vector. In Python 3.13.3, the “==” operator would normally have only the function of comparing memory addresses, but in PyCIFTer, it was replaced with comparison of the position vectors for two atoms.
- __hash__(self): defines what the hashing input should be when generating the hash of an atom. The position vector is unique to each atom, and, thus, it is used as a hashing input.

### 2.6. Manipulations of Atomic Coordinates and Structural Information

**Figure 6:**
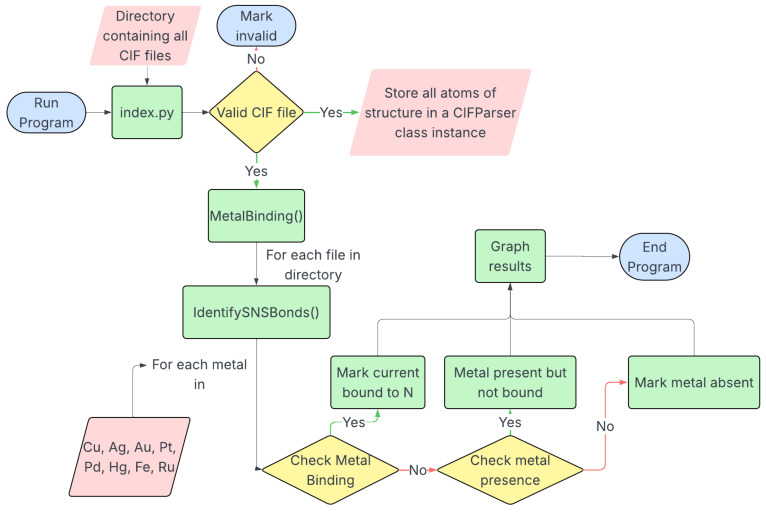
Diagram showing the workflow for analysis of structural data within the index.py file associated with PyCIFTer.

The bulk of the analysis work takes place in the index.py file and is split into multiple, different components as shown in Figure 6. The two main functions are **IdentifyingSNSBonds (ISB())** and **MetalBinding (MB())**. **ISB()** is responsible for generating the S–N–S bond graph, while **MB()** is responsible for (i) determining whether one or more metals are found in a given structure, (ii) determining whether the nitrogen of a given TFSI moiety is bonded to a metal, and (iii) processing the generated results into an appropriate format. The main program flow is in **MB()**, while **ISB()** is a utility function, albeit a major one. **ISB()** is responsible for returning the S–N–S bonds in the given molecule. Identifying the [S–N–S] core of each molecule of TFSI found in a given structure is very important, of course. This is because the identification of this core motif is needed to then identify whether the N atom contained in the core is bound to a metal. Because of the criticality of this step to the project, two individual approaches were considered, and then the better of the two was chosen for full implementation.

The first approach involved traveling and mapping out all bonds between every atom in a structure through a Breadth-First-Search (BFS) algorithm. A BFS algorithm is a graph-searching algorithm that works on a first-find/first-search basis [60]. The algorithm is executed by beginning at an arbitrary node in a graph and then sequentially storing all nodes connected to the original node in a queue. The next step involves processing each node on the queue in the order that they were placed into the queue. Then, the procedure is repeated by adding each new node’s neighbors into the queue. This procedure is fundamental to graph theory and is immensely useful in navigating graph problems [60]. After carrying out the first BFS-based mapping of all bonds, the program can initiate a second BFS from all the stored nitrogen atoms to determine all the S–N–S bonds. Such a method is quite robust and would account for all possible occurrences of the [SNS] motif.

However, charting the entire structure of every molecule in our rather large structure set (containing ca. 1500 individual CIF-format files) would have been computationally expensive and would be unnecessary in this context, as the motif of interest is relatively small. Therefore, instead of this first approach, we decided to go with a second possibility: a localized search to isolate individual [N–S] bonds in each structure. The process of mapping the S–N–S bonds in each structure with this approach is described below.

The first step in the chosen method for mapping the bonds of [SNS] moieties is to carry out a brute-force search through all possible combinations of N and S atoms in a given structure followed by calculation of the Euclidean distance between each pair of N and S atoms. The resulting distance value for each N/S pair is then compared to the covalent radii of the involved atoms (N and S, of course) using the following equation:

(5) $$distance\leq \left.\left(N.covalentRadius+S.covalentRadius+\tau \right.\right)$$

In this equation, distance is the standard Euclidean distance between each pair of N/S atoms, and the quantity τ represents a standard factor for determining whether a given separation between atoms is sufficiently tight to be considered a covalent bond. The value of this factor (τ) of 1 Å was used throughout this project after conducting an analysis examining differences in results as a function of the value of τ. We considered values of τ from 0 to 2 Å and found that 1 Å was sufficient for our purpose. For comparison, Sheldrick’s immensely useful program XP for molecular graphics utilizes a similar standard factor for determining if interatomic separations correspond to bonds. In XP, the value of the standard factor is 0.5 Å. As our value is greater, the stringency for determining if an atom-to-atom distance corresponds to a bond with our program is lower. Greater distances could be interpreted as corresponding to weaker bonds in our analysis. 

If the inequality in Equation (5) was satisfied, the given (N,S) pair was defined as bonded, and a tuple containing the atoms (generally, an immutable list of values; in the case here, a pair of Atom instances) was added into a HashMap titled **distanceValues**. Additionally, the number of occurrences of specific N atoms is tracked in a HashMap titled **occurrences**. The schemas of the HashMaps are as follows:

$$distanceValues=\left\{\left.\left(N,S\right.\right):\;<Distance\;in\;Å>\right\}$$

$$occurrences=\left\{N\;atom:\;frequency\;in\;distanceValues\right\}$$

The main motivation behind this algorithm comes into play at this point. We iterate through the **occurrences** HashMap to find N atoms with a frequency more than 2 and then find the corresponding bound S atoms from **distanceValues** to generate the [SNS] moiety. The following process then takes place for each [SNS] moiety in a molecule: each bond is then stored as an acyclic undirected graph and is represented using a graph adjacency HashMap with the following schema:

$$structure=\left\{Atom\;A:\;\left[List\;of\;all\;other\;atoms\;bound\;to\;A\right]\right\}$$

The structural orientation of the moiety is stored in a Graph class. The Graph class serves as an encapsulation for the [SNS] moiety, meaning that it is a way to represent the structural information of a particular [SNS] moiety with ease. Stated another way, the Graph class represents an abstraction of the [SNS] moiety. This class has instance variables for the adjacency HashMap structure, bond angle, and other utility functions.

Importantly, the S–N–S bond angle is calculated by applying vector geometry operations on the Graph class. The process involves generating two vectors, $v_{1},v_{2}$, which point to each of the S atoms from the N atom in the center. We then use the dot product vector operation and manipulate the equation to obtain the S–N–S angle and store it in the Graph object, as follows:

Let $v_{1}$ and $v_{2}$ be vectors pointing from N to S_1_ and from N to S_2_. Then the equation for the dot product of two vectors is:

(6) $$\vec{v_{1}}\cdot \vec{v_{2}}=\left|\left|\vec{v_{1}}\right|\right|*\left|\left|\vec{v_{2}}\right|\right|\cos\theta$$

After trivial rearrangement of the equation, one arrives at the following result:

(7) $$\theta =\cos^{-1}\left(\frac{\vec{v_{1}}\cdot \vec{v_{2}}}{\left|\left|\vec{v_{1}}\right|\right|\cdot \left|\left|\vec{v_{2}}\right|\right|}\right)$$

In this case, the value of *θ* is the bond angle between the relevant S, N, and S atoms of a given TFSI moiety about which information is stored in the Graph class. At this point, PyCIFTer concludes the process of abstracting the desired information for a single [SNS] moiety. And, at this point in the workflow, PyCIFTer will repeat the process for all [SNS] moieties in each input CIF/structure (as there can be multiple TFSI anions per structure). After this, PyCIFTer concludes the process of parsing a single CIF in the dataset. Along the way in wrapping up these steps, the Graph object for each [SNS] moiety is appended to a running list, which is ready for further operations following the conclusion of parsing all the files in our dataset.

As mentioned above, the Graph class is an abstraction of the [SNS] moieties extracted through the previous analysis. The Graph contains a HashMap that stores the adjacency list. The S–N–S bonds are represented as a special type of graph. In the language of computer science, we note that a graph is made of nodes and edges. In PyCIFTer, the nodes are the atoms, and the edges are the bonds between the atoms. The edges are designed to be bi-directional to emulate the behavior of the sharing of electrons in “real” (covalent) chemical bonds. Finally, we note that the [SNS] motif is, in a chemical sense, acyclic. There is not a continuous circle of atoms in the structure, but rather there is a set of three atoms that are merely adjacent and bonded to each other in the TFSI anion. Thus, and also in line with the language of computer science, the graph is called an acyclic undirected graph. The graph is acyclic here, owing to the nature of the atom adjacency in the chemical moiety being studied, and also undirected because of the envisioned bidirectional nature of the covalent bonding. The adjacency list is stored as a HashMap with the structure HashMap schema given on p. 14.

The Graph class also has utility functions defined below:

- addBond(self, existingAtom, newAtom, distance): adds a new bond between **newAtom** and existingAtom to the **structure** HashMap.
- returnAtoms(self, symbol): returns all the atoms of a specific element “symbol” in the **structure** HashMap.

The **MB()** function (vide supra) is the main site of program execution. This function calls on the **ISB()** function to retrieve all the abstracted [SNS] moieties and then proceeds to conduct operations on these moieties. The function begins with defining variables that are essential to storing the program’s running components. Additionally, these variables store a running count of the properties being investigated corresponding to each file, which are eventually passed to the graphing modules. The three most important variables that should probably be mentioned are the following:

- AnglePlotValues[]—a list of floating-point numbers that store the S–N–S bond angle for each moiety.
- bondLengthAverage[]—a list of floating-point numbers that store the average of the two individual N–S bond distances for each [SNS] moiety.
- fudgeFactor—a list of floating-point numbers from 0 to 3 in 0.1 increments that represent the range of τ values (vide infra) utilized for sensitivity analysis.

The function loops through each CIF in the directory containing the full input dataset. Each CIF path is passed as an argument into the constructor of the CIFParser class, and the resulting object is stored in a variable with the name **molecule**. The **molecule** variable is an instance of the CIFParser class and is intended to be a concise manner of handling all the characteristic properties of each CIF and the many atoms in each crystallographic structure.

The next step is to call the **ISB()** function to retrieve all the occurrences of the [SNS] moiety in a given molecule. The [SNS] moieties are stored as instances of the Graph class, and the ISB() function returns a list of these graph instances back to the **MB()** function. PyCIFTer iterates through the previously determined list of [SNS] moieties and their corresponding S–N–S bond angles, in order to, at this stage in the process, compute the related N–S bond length average. The bond length average for each [SNS] moiety is defined in Equation (8) as follows:

$$For\;a\;given\;S_{a}-N-S_{b},$$

(8) $$Bond\;Length\;Average=\frac{{(E}_{d}\left(S_{a},N\right)+E_{d}(S_{b},N))}{2}$$

where $E_{d}\left(A,B\right)=Euclidean\;distance\;between\;Atom\;A\;and\;Atom$.

The result of the above operations is then appended to the **bondLengthAverage[]** list mentioned at the start of the **MB()** section, and the bond angle is appended to **AnglePlotValues[]**.

The next steps are to (a) search for the presence of metallic elements in each CIF and (b) determine if a given metal is coordinated to the central nitrogen atom of each particular [SNS] moiety that was previously identified by PyCIFTer. Here, we note that coordination is defined here, in the chemical sense, as featuring the involvement of a direct M–N interaction in the structure and as judged on the basis of atom-to-atom distance. We also note that due to the specific scholarly interests of our research in this case, PyCIFTer was designed to only search for direct M–N coordination in the κ^1^-[N] mode (see Scheme 2). PyCIFTer investigates metal coordination of an individual [SNS] moiety in two steps. The first step is to search for general metal presence in the local vicinity of a given moiety. This is facilitated by the getAtomsInARadius(self, targetAtom, radius) utility function offered by the CIFParser class, where the items listed in the parentheses represent the parameters that are to be passed into the function. The function searches through all atoms in each molecule and returns a list of atoms such that the Euclidean distance between the **targetAtom** (in this case the central nitrogen of the given [SNS] moiety) and a compared atom (an arbitrary atom in the molecule) is less than or equal to the **radius** parameter. This utility function emulates a wave of **radius** Å being emanated around the particular nitrogen and returns a list of all atoms (called **surroundingAtoms**) that the wave encompasses. PyCIFTer searches for relevant metals iteratively by examining the **surroundingAtoms** list. (The group of metals of interest was determined with a preliminary analysis of the entire dataset of CIF-format files using a beta version of PyCIFTer. A metal was considered to be of interest if the frequency of its presence in a TFSI structure, either coordinated to an [SNS] moiety or not, was greater than or equal to one.)

The second step of the final analysis completed by PyCIFTer, step (b) above, involves cycling through the list of relevant metals and initiating a two-stage check on each relevant metal. The first stage of this step is to determine if the metal is coordinated to the nitrogen atom of an individual [SNS] moiety. In order to do this, tabulated covalent radius values from the literature [51] for the individual metals and nitrogen are utilized, in order to establish a threshold criterion for coordination. The mathematical equation for evaluating coordination is given below:

(9) $$distance\leq \left.\left(N.covalentRadius+M.covalentRadius+f\right.\right)$$

where

*distance* = Euclidean distance between the Nitrogen and Metal;

*N *The central nitrogen atom in the S−N−S moiety;

*M *One of the metals of interest.

If this condition is satisfied, the metal is considered coordinated to the nitrogen of an individual [SNS] moiety, and the particular [SNS] moiety in question is categorized as “Metal present and N-bound to TFSI.” If the condition is not satisfied, the program initiates the second stage of metal analysis to see if any metals of interest are present in the structure (but not within bonding distance to any identified TFSI (i.e., [SNS]) moieties). PyCIFTer determines if the current structure contains the metal atom of interest (as defined in this stage of the process) using the **containsAtom** function offered by the CIFParser class. If the complete structure represented by the CIF contains the metal, it means that either the metal was present within a 3 Å radius around the nitrogen of an [SNS] moiety but not directly coordinated to nitrogen or that the metal was present in the general compound at distance greater than 3 Å from the particular [SNS] moiety but, again, not coordinated to it. In this case, the [SNS] structure is marked as “Metal present but not N–bound to TFSI”. Otherwise, with both the stages yielding false values, a particular [SNS] moiety is categorized as “No metal present in the structure.”

### 2.7. Determination of Coordination and the Importance of the Tau Factor

The process of determining whether a metal is coordinated to a given [SNS] moiety involves emanating a wave or radius 3 Å to define a search region around the nitrogen atom of the TFSI anion. This region is used to retrieve a list of atoms the wave encompasses. Subsequently, PyCIFTer iteratively checks each element in the retrieved list of atoms and checks whether the inequality discussed above and shown in Equation (9) is satisfied. In the development process for PyCIFTer, the circular search geometry was chosen because such a geometry is straightforward to implement and scale to an arbitrary size. Secondly, by localizing the region where PyCIFTer searches for possible metals close to nitrogen to a radius of 3 Å, PyCIFTer considerably reduces its search space from (possibly) all the atoms in a given CIF to just the atoms present in the sphere of radius 3 Å. Consequently, this decision results in a drastic reduction in the computational complexity and time required by PyCIFTer. Lastly, the 3-Å radius aims to be an inclusive check covering any possible metal bond length that may appear in the dataset. Informed by experience with chemical structure and bonding across various metals and ligand systems, we anticipated that no relevant metal–ligand bond would exceed a distance of 3 Å. Thus, the chosen search radius of 3 Å could be safely concluded to be sufficient for recognizing any metals that are coordinated to the nitrogen atom of the [SNS] moieties.

In our view, a chemically sensible approach for bond identification involves an application of an inclusive criterion: the minimal distance for a bond to be considered would be the sum of the covalent radii of the nitrogen and the metal of interest (pulled from the covalentRadii HashMap), along with a variable parameter, τ. This approach has been taken before in prior programs used for crystallographic/structural analysis, such as Sheldrick’s XP as described on p. 13. Such an approach accommodates bonding patterns where metal cations are coordinated to the nitrogen of a TFSI moiety, even if the intrinsic chemical characteristics of the molecular species of interest result in structural properties that drive a nitrogen–metal bond length significantly longer than the previously mentioned minimum distance, which would be the sum of the literature covalent radii stored in the covalentRadii HashMap.

To implement the approach described above, the variable parameter τ was included in the program, and, as described in the general discussion of the program above, it served as a “fudge factor” of sorts that the user of the program could adjust in order to set a sliding stringency scale for the determination of the N-coordination of a metal to a TFSI moiety. Having declared τ in PyCIFTer, we, as users, could go about a straightforward sensitivity analysis in order to define a sensible and statistically validated value of τ to implement in the final build of PyCIFTer (see Figure S1 in the Supporting Information). This process of the optimization of τ was, of course, facilitated by the rapidity with which PyCIFTer could be quickly and iteratively run on the entire dataset of 659 CIF-format files containing 1122 individual TFSI anions.

Along this line, during development of the final version of PyCIFTer, over the course of one hour we repeatedly ran the program on the entire dataset with various τ values ranging from zero to three in 0.1-Å increments in order to arrive at the final value that we anticipated would be generally suitable for the purposes of our investigation. In this case, as described above, our program’s purpose was an investigation of coordination of any metal with the nitrogen of the TFSI anion. In our view, it was desirable to make the process of coordination determination as inclusive as possible. Thus, the final value of τ that was selected is 1 Å. This choice means that even quite long metal–nitrogen distances are classified by PyCIFTer as putative bonds. A summary of the results of the sensitivity analysis is shown in Figure S1, indicating that a value of the floating parameter of 1 Å is sufficient to capture nearly all putative metal–nitrogen interactions of interest.

### 2.8. Graphing the Results for Quick Review

PyCIFTer employs the open source graphing package Matplotlib [61] for the visualization of raw data through computer-generated plots. An example of such a plot is shown in Figure S2. PyCIFTer supports generating scatter plots, interactive live scatter plots, histograms, and bar graphs. Additionally, PyCIFTer offers a GitHub repository [62] and a dedicated dashboard website, which leverage Plotly Express [63] to generate and display interactive graphical representations of the analytical results. Lastly, PyCIFTer uses the xlsxwriter [62] Python module to optionally generate Microsoft Excel (xslx-format) sheets containing raw graphing data. The authors emphasize that all of these functionalities were used during the development of PyCIFTer, as well as during final analysis of the structural data, in order to survey results and draw conclusions based on the data.

### 2.9. Results of Structural Analysis and Findings Regarding TFSI Properties

Once the final version of PyCIFTer was completed, it was packaged for public distribution into the version that is now available on Github [62] and utilized by us to carry out the desired analysis on the coordination properties of the TFSI anion. Using the 2024.3 update to the Cambridge Structural Database, 659 CIFs for TFSI-containing species were used as the input dataset for analysis by PyCIFTer. Importantly, as described above, only structures lacking disorder were used here, in order to simplify considerations around distinguishing between disordered components of the anion and/or disorder between individual molecular fragments containing metals. Certainly, an opportunity for further development would be program improvements that support the inclusion of disordered structures. This is the case both from the standpoint of the desirability of a complete analysis of all available data and from the standpoint of program development to address the often-encountered issue of disorder in solid-state structures more broadly. Inclusion of appropriate treatments of space group symmetry would also be desirable in future versions of the program (vide supra). However, due to the intrinsic structural flexibility of TFSI, we anticipate that there would be few cases in which TFSI moieties are located on or near rigorous symmetry elements.

Within our dataset of 659 input CIFs, 1122 individual [SNS] moieties within TFSI anions were identified by PyCIFTer for analysis. Of these moieties, 366 were found in structures that also contained a metal somewhere in the unit cell; 756 were found that do not contain any metals (see Figure S4). Considering that 67% of crystallized TFSI anions were found in structures that do not contain metals, it is clear that metals are not required for TFSI crystallization. Indeed, the majority of work with TFSI from a structural chemistry perspective has been achieved without involving metals. At this stage, we anticipated that this was the case due, in part, to interest in the use of TFSI as an anion for supporting the development of organic materials such as ionic liquids and electrolytes. Metal cations are neither necessary nor desirable for inclusion in such applications, and indeed, the use of organic countercations would be desirable for applications of TFSI species in ionic liquids. However, we note here that the apparent preference for the crystallization of diffraction-quality crystals lacking metals could be attributable to other effects. For example, based on the available data, it appears that species containing metal TFSI salts may be resistant to crystallization. Furthermore, as we omitted disordered structures from our analysis, it might also be the case that the presence of metals induced disorder that biased the available data in the observed direction. Perhaps consistent with these possibilities, a search of the CSD for the TFSI moiety including all structures (thus not excluding disordered structures) returns a total of 1394 hits (as shown in Figure 1). Thus, less than half of TFSI-containing structures are non-disordered. This suggests that the TFSI anion and the complexes and materials that it can be used to generate are quite prone to disorder. This is chemically sensible given the flexible nature of the chemical structure of TFSI, which, of course, we were able to investigate here using our geometric analysis with PyCIFTer.

**Figure 7:**
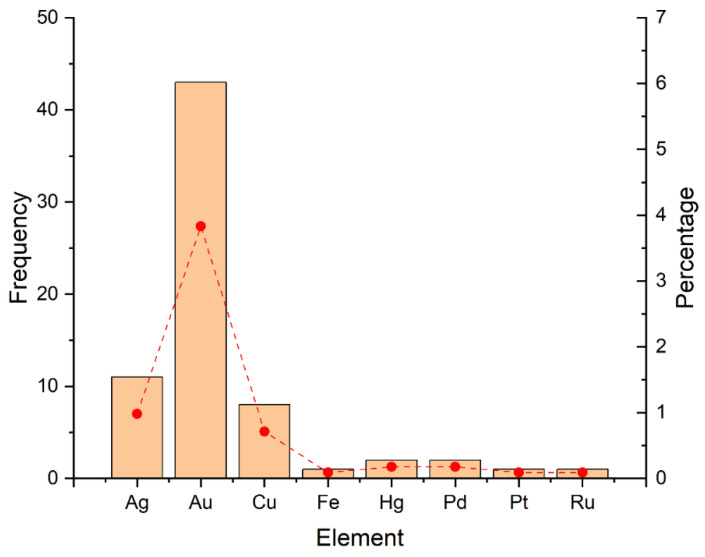
Results of metal coordination analysis. The left y-axis, associated with the bars shown for each element, denotes the raw frequency of hits of [SNS] moieties coordinated to metals. Each bar corresponds to the individual number of metal-bound moieties with the noted metal/element names. The right y-axis (associated with the red points connected by dashed lines) denotes the percentage of [SNS] moieties coordinated to the individual metals with respect to the entire [SNS] moiety count.

The 366 TFSI-derived [SNS] moieties that were found by PyCIFTer to be associated with structures also containing metals correspond to 33% of the total number of moieties interrogated. Among these, a total of only 69 [SNS] moieties were found to be coordinated via nitrogen to a metal. Thus, only 6.1% of the crystallographically characterized individual TFSI anions were N-bound to a metal. This total is broken down by metal identity in Figure 7. Gold is the most prevalent in this group with 43 individual moieties; silver and copper are next with 11 and 8 moieties, respectively. There are two N-bound moieties for mercury and palladium, and just one for iron, platinum, and ruthenium. In our view, this data suggests that a reliable conclusion is that very few of the crystallographically characterized TFSI moieties are N-bound to a metal. Looking at the data in terms of the total number of structures (rather than the number of individual, crystallographically defined moieties), there were 659 input CIF-format files used for our analysis. Of these, 238 (or 36%) contain metals in the structure but only 55 (or 8.3%) contain one or more metal centers that are N-coordinated to TFSI. N-coordination of TFSI to any metal can thus safely be concluded to be only uncommonly observed and, so far, only observed for transition metals.

The viewpoint that N-coordination is only rarely observed was confirmed by conducting several individual manual searches of the CSD in order to identify different coordination modes. In line with the results from PyCIFTer, 59 individual CIF-format files/structures were found when searching for metal cations N-bound to TFSI, a figure that aligns well with our program’s result that 69 individual TFSI moieties feature N-bound cations. This result also suggests that among the 55 individual structures containing TFSI anions, several must contain multiple crystallographically unique and defined TFSI species. This is not surprising given the recognized ability to prepare stable salts of mono-, di-, and trivalent metals featuring TFSI counteranions [32]. The process of obtaining these results and comparing them to manual search results suggests harmony between the PyCIFTer results and those that can be obtained manually by conventional querying with the ConQuest program. However, as noted above, PyCIFTer carries out the analysis rapidly, whereas manual searching and analysis of the results takes significantly more time (hours to days, in our experience). PyCIFTer was designed to both execute a rapid search for particular elements that are associated with TFSI moieties in crystallographic data and rapidly analyze multiple geometric properties of the [SNS] core motifs found in the TFSI anions. These geometric properties include the average S–N distance in individual [SNS] moieties and S–N–S bond angles. (Capability for calculating torsion angles is also present in the program, but as no meaningful trends in the particular dataset used here were found, and no results generated with this aspect of the program are discussed here.) In this sense, PyCIFTer is more specialized than a general query of the CSD for crystallographic results and can also more rapidly output geometric data than conventional approaches.

**Figure 8:**
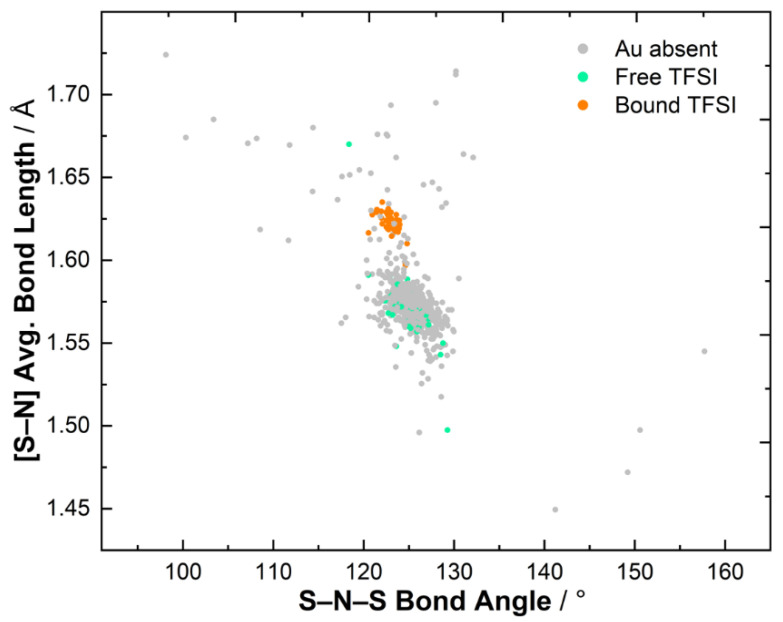
Plot focusing on results for gold cations showing the distribution of structures as functions of S–N–S angle and average S–N bond length. The orange points correspond to [SNS] moieties that are N-bound to gold, the light green points correspond to [SNS] moieties that were found in structures that contain gold atoms (but in which the copper is not N-bound to a TFSI moiety), and the gray points correspond to [SNS] moieties that are found in structures that do not contain gold.

**Figure 9:**
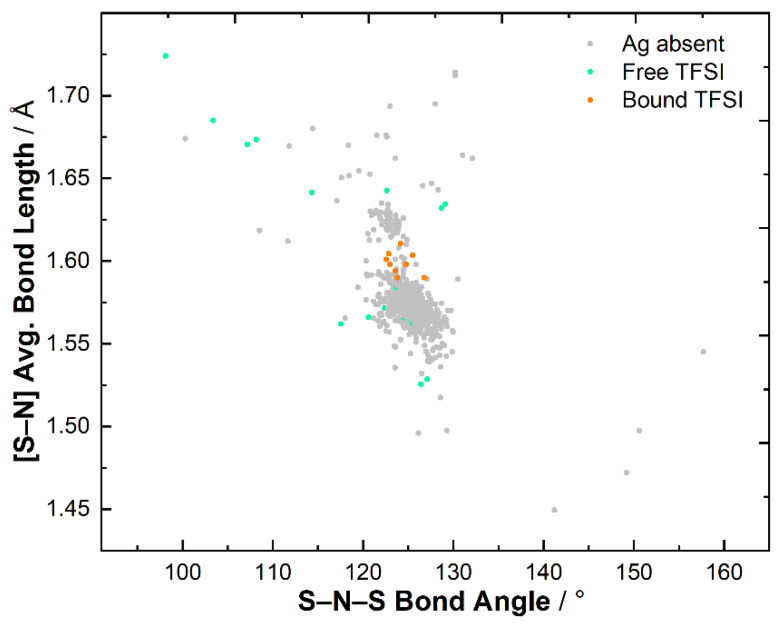
Plot focusing on results for silver cations showing the distribution of structures as functions of the S–N–S angle and average S–N bond length. The orange points correspond to [SNS] moieties that are N-bound to silver, the light green points correspond to [SNS] moieties that were found in structures that contain silver atoms (but in which the copper is not N-bound to a TFSI moiety), and the gray points correspond to [SNS] moieties that are found in structures that do not contain silver.

In order to examine the metal-specific findings shown in Figure 7 in more detail, data were plotted for all of the individual [SNS] moieties found in the chosen dataset as functions of two geometric parameters calculated by PyCIFTer, the S–N–S angle, and average S–N distance. The results for gold are shown in Figure 8, results for silver are shown in Figure 9, and results for other metals are shown in Figures S5–S10. In Figure 8, the orange points correspond to [SNS] moieties that are N-bound to gold, the light green points correspond to [SNS] moieties that were found in structures that contain gold atoms (but in which the gold is not N-bound to a TFSI moiety), and the gray points correspond to [SNS] moieties that are found in structures that do not contain gold. Thus, the points together correspond to the total of 1122 [SNS] moieties found by PyCIFTer for analysis. Considering that S–N average bond length and S–N–S bond angle are plotted on the x- and y-axes, respectively, Figure 8 demonstrates a number of trends. First, the great majority of TFSI moieties that were analyzed feature S–N bond lengths and S–N–S angles that are clustered in a hot spot that is centered near a bond length of 1.58 Å and a S–N–S angle of 125°. This cluster contains both structures featuring gold and also lacking gold, which is sensible. Considering this, it becomes apparent that the natural structural preference for TFSI is to adopt an S–N–S angle of around 125° and a S–N bond length of 1.58 Å. This distance appears reasonable given comparison to the standard tabulation of N–S distances that is available in Volume C of the *International Tables for Crystallography* and based on a comprehensive analysis of available solid-state data [64]. For example, N•••S bonds in moieties of the types [N=S=N] and [N=S=S] have an average distance of ca. 1.54 Å. And N•••S bonds in [C–SO_2_–NH_2_] have an average distance of 1.60 Å. (Listings for the noted sulfur-containing moieties are given on p. 809 of [65]). The [S•••N•••S] moiety in the TFSI anion would be expected to have a somewhat longer bond than the noted classes of multiple bonded literature moieties, and perhaps a bit shorter than the amino-sulfonyl class of species. The bond angle of 125° is also sensible given the anticipated quasi-sp^2^ hybridization of the nitrogen atom in TFSI; this angle being greater than an idealized 120° is likely reflective of dual influences of the possible steric clash between mutually oriented trifluoromethyl groups. Charge delocalization from the formally anionic nitrogen centers by the electron-withdrawing [SO_2_CF_3_] functionalities could also influence the S–N–S angle as well.

Second, there is an obvious additional hot spot in the data centered near a similar S–N–S bond angle but at a significantly longer S–N bond length of ca. 1.625 Å. This second hot spot is associated with the TFSI moieties that were found that are N-bound to gold metal atoms. Consequently, extraction and plotting of this data gives rapid access to the notable conclusion that N-coordination of TFSI to gold (and other select metals, vide infra) results in significant deformation of the [SNS] core motif of TFSI away from its native structural preference that is reflected in the apparent primary hot spot. Noticeably, this second hot spot in the data is also observable for the case of silver cations, although the elongation of the average N–S bond is not as significant in comparison with gold.

**Figure 10:**
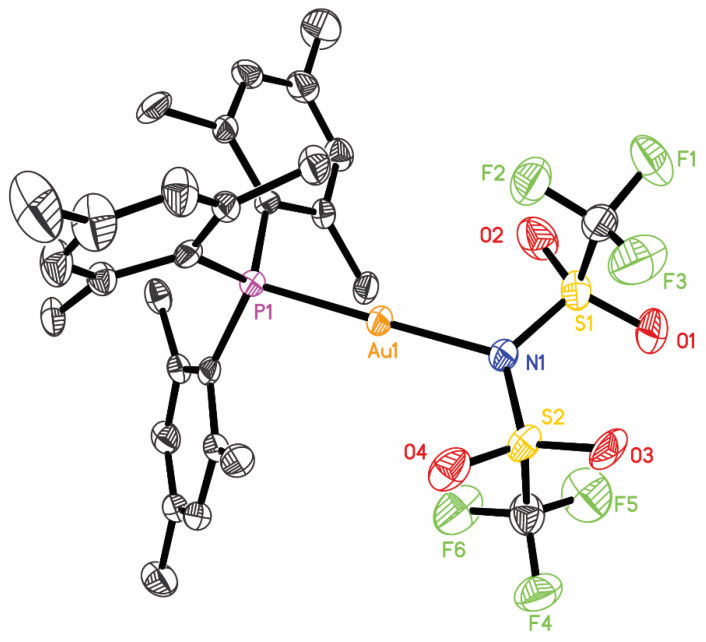
Solid-state structure from XRD analysis in which one TFSI anion is coordinated to a gold complex in the κ^1^-[N] mode (through nitrogen atom N1). All hydrogen atoms are omitted for clarity, and displacement ellipsoids are shown at the 20% probability level. From references [65,66].

An example of a structure associated with the gold-bound hot spot in the structural data is shown in Figure 10. The structures in the hot spot, like the example shown in Figure 10, all feature two-coordinate gold centers; one ligand to the gold center in each of the structures is a κ^1^-[N]-bound TFSI anion and the other is a neutral donor ligand. Thus, the unique geometries and structural properties of the TFSI anions in the second hot spot appear to be associated with gold complexes that are two-coordinate. In these structures, the nitrogen of the [SNS] core motif of TFSI is found in a trans position relative to the donor atom of the second ligand. The second ligand, like TFSI in a sense, tends to be sufficiently bulky to disfavor binding of other ligands to the metal center; in the structure shown in Figure 10, the second ligand is trimesitylphosphine (TMP). TMP features methyl groups positioned appropriately to help enforce the two-coordinate structure of the complex in this case. Notably, the formation of the two-coordinate structures is consistent with the measured lengthening of the average S–N bond distance in the coordinated TFSI anion relative to most other crystallographically characterized TFSI anions analyzed in our study. Coordination of a lone pair associated with the nitrogen donor atom of the TFSI anion to Lewis acidic Au(I) diminishes the availability of electron density from nitrogen for donation to sulfur, disfavoring the N–S multiple-bond character and thus driving the observed bond elongation. The data shown in Figure 9 for silver reinforce this conclusion; as a second-row transition metal, silver can be anticipated to engage in less significant covalent bonding with nitrogen of N-bound TFSI, resulting in a less significant modulation of the N–S multiple-bond character in the silver structures featuring N-bound TFSI.

### 2.10. Transferability: Potential for Applying PyCIFTer to Other Structural Motifs

Reviewing what was already discussed above, PyCIFTer can be broken down into two distinct parts: the CIFParser class and the index.py module. The CIFParser class is responsible for parsing an input CIF and creating a programmatic representation of a CIF unit cell that will be used by the index.py module. Index.py is the site of the computation necessary in identifying the [SNS] moiety and determining the presence of metals and whether the metal is bounded or not in each unit cell. By design, these two modules within PyCIFTer were designed to be two components of a system that could, in principle, be decoupled. In other words, the calculations and operations in index.py are not dependent on the source code written in the CIFParser class. The functions in index.py just use the high-level helper functions (vide supra, the functions are mentioned on page 12 of the manuscript) provided by the CIFParser class without any knowledge of the underlying source code. This deliberate design choice allows programmers to build custom Python scripts (taking inspiration from index.py) to analyze any moiety of interest. Hence, a key advantage of PyCIFTer lies in its integral modularity; with only minimal editing of the source code, diverse and expansive analyses could be conducted on a variety of triatomic motifs of interest.

To put PyCIFTer’s modularity into perspective, the [N(SO_2_)_2_] core found in TFSI could, in principle, be studied within a broader dataset that contains all structural data featuring an [N(SO_2_)_2_] core. Extension of the analysis on TFSI reported here to this broader structural series would require no changes to the source code to be accomplished. Of course, this broader dataset represents a dramatic increase in the number of potential structures for analysis, from ca. 650 for TFSI-containing structures to 2465 for all structures that contain an [N(SO_2_)_2_] motif. Further interrogation of these 2465 structures revealed some unique “TFSI like” moieties. For example, 2351 structures contained a [N(SO_2_C)_2_] fragment, 589 structures contained a [N(SO_2_C_2_)_2_] fragment, and 58 structures contained a [N(SO_2_F)_2_] fragment. The complexity of the molecular motifs containing just the N(SO_2_)_2_ fragment shows that PyCIFTer could be quite useful for continued exploration of this chemical space.

**Scheme 3:**
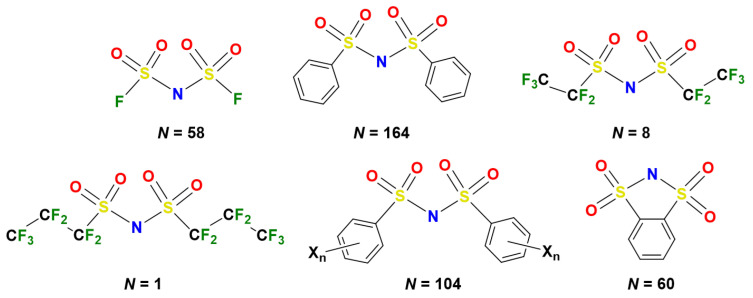
Diagram of some of the most prevalent “TFSI like” motifs found in the CSD. *N* denotes the number of structures that contain each respective motif. For the phenyl-(SO_2_) motif, X_n_ denotes any halogen-containing species wherein one structure was *meta* disubstituted (X_2_), one structure was perhalogenated (X_5_), and 102 structures were *para* monosubstituted (X_1_).

Although detailed structural analysis on motifs other than TFSI is outside the scope of this work, we did conduct a series of searches (using the CSD Conquest program) for molecular structures that contain the “TFSI like” characteristic of having particular [N(SO_2_)_2_] cores. In this series of searches, we found that there are distinct classes of molecular structures that feature a variety of terminal groups on the sulfonyl core. Some of the most prevalent of these moieties are shown schematically in Scheme 3.

As was found in the case of TFSI, we observed unique coordination modes of the various [N(SO_2_)_2_]-containing species with metal centers. For example, in structure EZIYIX [67], the [N(SO_2_)_2_]-containing species is coordinated in the κ^1^-[N] mode to Ag with a Ag1•••N1 bond distance of 2.204(2) Å. In structure LEBLIN [68], the [N(SO_2_)_2_]-containing species is coordinated in the κ^2^-[O] mode through both O6 and O9 to K1. The bond distances between the two coordinated O atoms, O8 and O9, and K1 in this case, are sensibly similar at 2.838 and 2.845 Å, respectively. And finally, in COHFAK [69,70], the [N(SO_2_)_2_]-containing species is coordinated in the unusual κ^2^-[N,O] mode through N1 and O1 to Cu1. In this case, the N1•••Cu1 distance of 2.006(2) Å indicates strong coordination, whereas the O1•••Cu1 distance of 2.626 Å seems to represent a weaker electrostatic interaction with the copper center.

**Figure 11:**
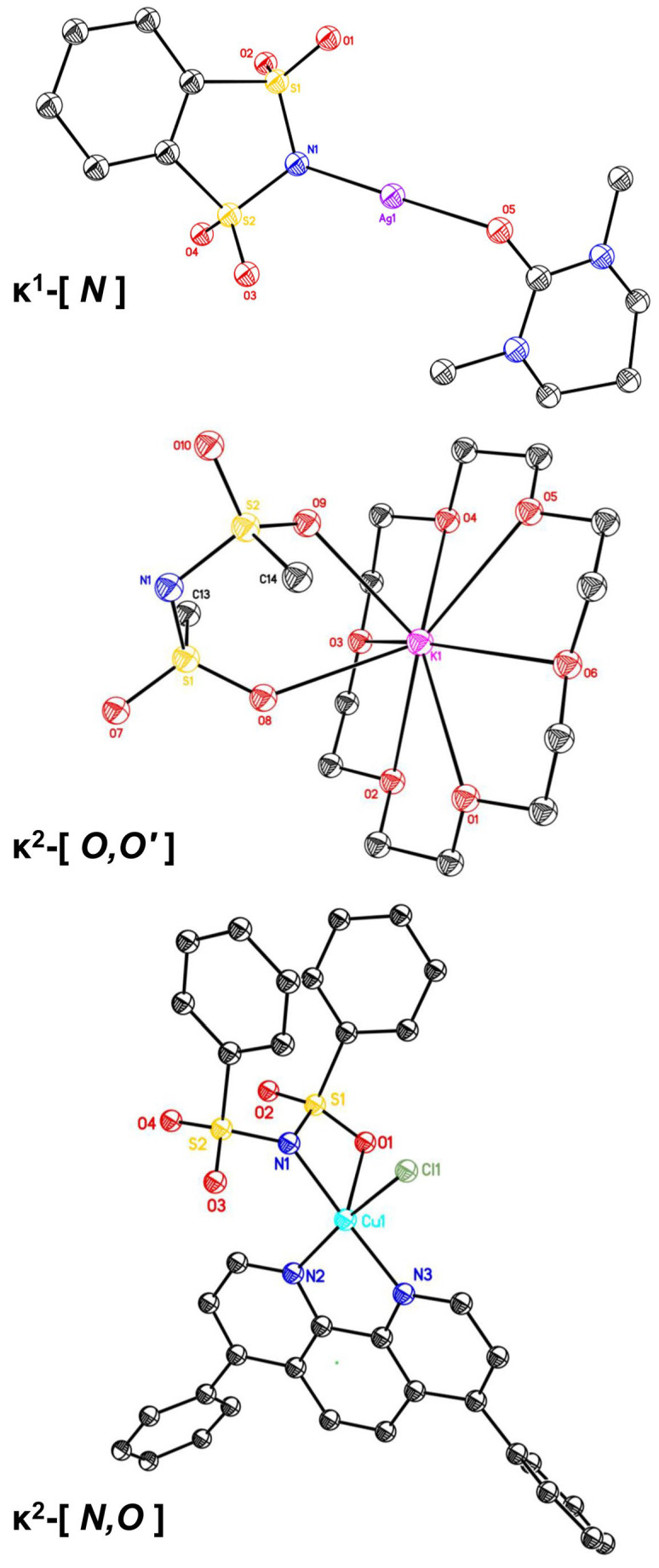
Solid-state structures of representative “TFSI like” anions participating in inner-sphere coordination with metal centers. Left: κ^1^-[N] bound (through N1 to Ag1); middle: κ^2^-[O] bound (through O6 and O9 to K1); right: κ^2^-[N,O] bound (through N1 and O1 to Cu1). All H-atoms have been removed for clarity, and atoms are shown with general isotropic displacement ellipsoids. From references [67,68,69,70].

In the case of the structure shown as the topmost structure in Figure 11, κ^1^-[N] coordination features a unique heterocyclic structure containing the N(SO_2_)_2_ core substituted onto adjacent positions of a benzene ring. We anticipate that the structural rigidity of the sulfonyl moiety may favor the observed κ^1^-[N] binding in this case. The structural rigidity of this motif is distinct from the high flexibility of the TFSI motif, wherein the trifluorosulfonyl terminal groups have free rotation around the central nitrogen and the trifluromethyl groups also have free rotation, presumably leading to a diverse range of available coordination geometries.

A more direct comparison to TFSI is found in the middle structure of Figure 11. This structure contains the [N(SO_2_)_2_] core appended with terminal methyl groups. This counteranion seems uniquely poised to engage in κ^2^-coordination via the sulfonyl oxygens for two reasons. First, the absence of trifluoromethyl terminal groups increases the available electron density for donation from oxygen to a metal center (such as K). Second, the ability of the flanking sulfonyl groups to rotate allows for a suitable geometric match in this example structure.

A rare example of κ^2^-[N,O] coordination can be seen in the lower structure in Figure 11. To the best of our knowledge, no single-crystal structures containing TFSI exhibit this coordination mode, although it is theoretically imaginable (compare with the other modes shown in Figure 2). One can imagine that this is mostly due to the inherently weakly coordinating nature of TFSI, as direct coordination with metal centers is observed in only a few cases; most structures contain TFSI in the outer sphere. Of note here, the electron-withdrawing nature of the trifluoromethyl groups likely impedes κ^2^-[N,O] coordination due to the strong delocalization of the lone pair of electrons away from both the central nitrogen and the sulfonyl oxygens. However, in the example shown in Figure 11 that features terminal phenyl groups on the [N(SO_2_)_2_] core, both inductive and resonance stabilization of the lone pair on nitrogen and oxygen could contribute to the uncommon coordination mode in this case.

To complement the above analysis and further demonstrate the transferability of PyCIFTer, we conducted a preliminary structural analysis on the bis(fluorosulfonyl)imide moiety, [N(SO_2_F)_2_]. This moiety is also referred to as FSI. Because of the similarity of the FSI motif to the TFSI motif, no change in the source code of PyCIFTer was required to execute an analysis in this case. The results show that out of the 49 non-disordered FSI-containing structures that were found in the CSD, seven of them have a metal bonded to the nitrogen center of the S-N-S moiety of FSI. And 19 of these structures have a metal present but not bound to the FSI moiety. These preliminary results underscore that FSI, like TFSI, is a rather weakly coordinating anion that tends not to bind to metal cations. This conclusion is in accordance with the similarity of the fluorinated TFSI and FSI structures.

As described above, PyCIFTer searches for all occurrences of the S–N–S moiety in each CIF provided as part of the input dataset and conducts a structural analysis to determine several properties. The index.py module packaged with PyCIFTer was designed to modular and, indeed, is suitable for largescale analysis of different R–X–R styles moieties. PyCIFTer’s software architecture was designed with the interdisciplinary nature of its application in mind. This foresight is reflected in the ease of customization of PyCIFTer, especially to analyze R–X–R structures.

We will now highlight the changes necessary to analyze two additional moieties adhering to the R–X–R structure: C–N–C and F–C–F. The IdentifySNSBonds() function has two list variables, centralAtoms and edgeAtoms. The centralAtoms list is used to store all occurrences of the central atom of a R–X–R moiety; i.e., X in each CIF structure and edgeAtoms variable, all occurrences of the R atom of a R–X–R moiety are stored. The distances between the centralAtoms and edgeAtoms are compared to determine possible bonds between R–X.

**Figure 12:**
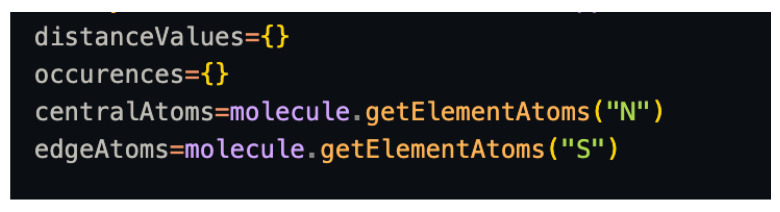
Snapshot of the lines of code from PyCIFTer that are associated with the key atom designations “N” and “S” that enable searching for the [SNS] motif within TFSI. When these atom designations are changed, searches for other motifs can be carried out with ease.

As seen in Figure 12 below, both centralAtoms and edgeAtoms are computed using the **getElementAtoms** helper function offered by the CIFParser class. Thus, with this level of robust implementation, one would only need to change the arguments (atom designations) placed into **getElementAtoms** to recognize C–N–C and F–C–F. To identify C–N–C bonds, one can set the element symbol argument for the centralAtoms **getElementAtoms** call argument to be “N” and the edgeAtoms **getElementAtoms** call argument to be “C”. To recognize F–C–F, the argument passed to the **getElementAtoms** call for centralAtoms should be “C”, and the argument passed to the **getElementAtoms** call for edgeAtoms should be “F”. By making the described changes, the index.py module could accurately recognize either CNC or FCF moieties.

In principle, PyCIFTer could have been applied to all the systems described above that contain [SNS] triatomic core motifs. Only minimal changes in the original source code would be required to execute analyses. However, it should probably be mentioned that PyCIFTer is not limited to only examples like those described above. The groundwork has been laid to expand applications of PyCIFTer to the widest possible variety of triatomic motifs of interest, including CNC, CNN, NNN, NCN, NCP, and others. To provide some context, a simple CSD search for a [C–N–C] fragment bound to any metal center via the central nitrogen identifies nearly 350,000 distinct entries in the database. Expanding the use of PyCIFTer to studies of CNC-containing ligands could provide immense quantitative insights. These and others are now waiting to be discovered with PyCIFTer and other programs available for large-scale structural analyses to be executed.

### 2.11. Comparison of PyCIFTer to the CSD Python API

The CSD Python API is a programming interface that provides direct, programmatic access to the Cambridge Structural Database (CSD) [24,25]. As described above, the CSD is a comprehensive and curated repository of small-molecule organic and metal–organic crystal structures. As a component of the licensed CSD Portfolio, the API is designed for chemical and data science applications, allowing researchers to automate complex search, retrieval, and analysis tasks. Instead of manually using graphical tools like ConQuest or Mercury, the API enables one to write Python scripts to find structures based on substructural fragments, metadata, unit cell parameters, and other advanced criteria, providing a powerful foundation for large-scale statistical studies and data-driven discovery.

The API works by connecting directly to a proprietary, pre-parsed, and highly optimized database that is managed by the Cambridge Crystallographic Data Centre (CCDC). The API does not work by enabling users to parse raw CIFs. This mechanism makes searching and data retrieval exceptionally fast, a key advantage. When a query is executed, the API returns Python objects that are fully populated with the data corresponding to a particular structure. The flexibility of the API extends far beyond simple searching; one can use the API to calculate many geometric parameters (viz., bond lengths, angles, torsions), analyze intermolecular interactions and crystal packing, and even perform statistical validation by comparing the geometry of a given fragment to millions of others in the database using the built-in GeometryAnalyser module. This allows for the creation of sophisticated analysis pipelines, integrating CSD data with other Python libraries like pandas and matplotlib.

In the context of the availability of the CSD Python API, we anticipate that perhaps the most significant advantage of PyCIFTer is its contribution to open and directly verifiable science. Inherently, the CSD Python API is a proprietary, commercial product. Any research that relies on it is thus restricted by a paywall. Results obtained with the CSD Python API cannot be verified, reproduced, or built upon by researchers who do not have access to an up-to-date license. PyCIFTer, by being a custom-built tool, is open source by nature and is being shared in its full form alongside this publication [62].

Our program could perhaps be considered more flexible than the CSD Python API because it operates independently of any particular database. The CSD Python API does not parse raw CIFs, but rather it only reads the CCDC’s own pre-compiled, proprietary database format. Our program, in contrast, is a true parser. It can ingest and analyze any valid Crystallographic Information File (CIF) from any source. This allows one to perform analyses that are impossible with the CSD Python API, such as directly comparing statistical trends across data obtained from different major databases like the CSD and Inorganic Crystal Structure Database (ICSD) [71]. Comparing across databases can enable researchers to check if candidate chemical insights are universal or an artifact of biases that might affect one particular dataset. ICSD also provides programmatic access to inorganic crystallographic structures, but this feature is also licensed and not compatible with the CSD Python API. As a result, a researcher must independently analyze structures from the ICSD and CSD with two different commercial Python APIs. Our program PyCIFTer allows for simultaneous analysis of any results for which CIF-format files are available.

In other words, PyCIFTer can be distinguished by its ability to support data independence. Data independence, in this context, directly unlocks a broader scope of scientific inquiry. The CSD, by its own charter, is limited to organic and metal–organic structures. PyCIFTer can be pointed at the ICSD (Inorganic Crystal Structure Database) to analyze purely inorganic materials—like minerals, zeolites, and complex oxides—which the CSD Python API seemingly cannot directly access. Furthermore, PyCIFTer could be used to bridge the critical gap between experimental and theoretical chemistry. It can parse the theoretical CIFs generated as output from quantum chemistry simulations (e.g., Gaussian, VASP, Amsterdam Density Functional). This flexibility could enable researchers to directly compare the predicted geometries from computation against the experimental trends from single-crystal diffraction-based databases.

Stated another way, our program is a “white box”, whereas the CSD Python API is a “black box” to some degree. PyCIFTer offers complete transparency and control over the data analysis pipeline, but when one asks the CSD Python API for data analysis, one implicitly relies on internal, proprietary algorithms from the CCDC in order to interpret complex or ambiguous data. Ambiguous data, in this context, could include database entries with significant structural disorder, partial site occupancies, or non-standard formatting. However, with PyCIFTer’s open source nature, a researcher has complete control over the functioning and analysis of the program. This means our methodology is fully transparent, auditable, and defensible, as reviewers can inspect and validate every single step of the process if desirable.

At this stage, we anticipate that PyCIFTer, at least, represents what could be considered an open access analogue to other commercial programs like the CSD Python API. We note that, generally speaking, most of our own research work in synthetic, molecular chemistry is best carried out by using the powerful tools offered by the CCDC, including the CSD and the visualization program Mercury. Hence, it is our hope that specialized, open access tools like PyCIFTer can be used to accelerate scientific discovery alongside more traditional resources like the CSD and next-generation tools like the CSD Python API.

### 2.12. Outlook for Future Work

The development of PyCIFTer highlights a number of opportunities and challenges in the development of software tools for semi-automated analysis of structural data from X-ray diffraction analysis. First, use of this approach to gain insights into the properties and behaviors of particular molecular species can be viewed as attractive based on the satisfactory set of results obtained here. For example, in the course of our study, we learned that gold and, to a lesser extent, silver, tend to promote formation of N-bound TFSI species. This is a sensible result, but was not readily apparent to us at the outset of our study. Second, the speed of individual analysis runs with PyCIFTer could be viewed favorably in certain applications when compared to conventional ConQuest querying. The application explored here, repeated batch analysis of a large number of structures to extract structural/bonding parameters, is particularly effective in comparison to conventional querying. Conventional querying of the CSD has been among the most useful tools in our research program for examining prior work and simulating creativity around future projects, but we anticipate that semi-automated batch analyses can complement other approaches to use of the CSD. From the standpoint of challenges, we anticipate that the most significant barrier to more effective utilization of programs like PyCIFTer is crystallographic disorder. This is because of the inherent challenge in distinguishing the atoms associated with individual molecular species or isomers in solid-state data featuring disordered species; without a reliable approach to identifying the individual molecular species, erroneous results would certainly arise. Of course, there are approaches to dealing with disorder in existing software for crystallography, such as the SHELX system, and these could be used as inspirations for addressing the limitation here. In SHELX, PART instructions can be used to distinguish, for example, distinct and defined species that are co-crystallized in the same volume of the asymmetric unit. The ultimate solution to dealing with disordered structures in PyCIFTer could involve leveraging details from refinement models, like PART assignments, to focus on the analysis of appropriate atoms in disordered structures.

In any case, we anticipate that the outlook is bright for the use of programs, like the one described here, to accomplish demanding analyses in chemistry. The rich data contained in resources like the Cambridge Structural Database (CSD) are exceptionally useful for drawing conclusions about the behavior of individual chemical species, and also forecasting properties and behaviors that might be accessible in yet-to-be-prepared systems. Extrapolation of the patterns needed to make these forecasts can often be performed through chemical intuition and conventional one-by-one querying, but some applications are specialized enough and could draw on sufficiently large numbers of individual structures to motivate the development of tools like PyCIFTer. As featured in this paper, successful development of new tools is an interdisciplinary endeavor and one that was facilitated in this case by expertise spanning chemistry and computer science. Continued collaboration across these realms will no doubt accelerate further development of tools and afford exciting new strategies for the utilization of databases containing rich findings from prior research to contemporary problems.

## 3. Materials and Methods

The bulk of the analysis described in this report took place on a standard Apple Macbook with an M2 Apple silicon processor and 16 GB of RAM and 256 GB of storage. PyCIFTer was run on Python 3.13.3 with Matplotlib 3.10.1, Plotly 6.0.1 and Xlsxwriter 3.0.2. The Cambridge Structural database (version 2024.3) was accessed using ConQuest, and structures were viewed in three dimensions using Mercury. Using the noted Apple Macbook and the final version of PyCIFTer that is described here and available on Github [62], the full analysis of all 659 structures was found to be typically completed within less than five seconds.

PyCIFTer is a custom-built computer program. Unlike the CSD Python API, which is an external service that users can access using Python, all of the source code for PyCIFTer was built in-house. PyCIFTer uses standard python libraries, **os** and **io,** to read and write files, along with a variety of standard python libraries used for graphing and exporting data. The next few paragraphs highlight the packages and how they were used in the study.

NumPy (Numerical Python) is the fundamental package for scientific computing with Python. Its core contribution is the N-dimensional array object, or ndarray, which provides a high-performance, efficient container for large datasets and matrices. It enables vectorized arithmetic operations, which perform significantly better than standard Python loops for numerical tasks. NumPy serves as the foundational library for the scientific Python ecosystem, and, in this work, it was used specifically to simplify the process of computing a dot product and calculating angles between three atoms.

Matplotlib is a comprehensive plotting and data visualization library for the Python programming language [61]. It provides a robust, object-oriented API for creating a wide variety of static, animated, and interactive visualizations. Its pyplot module offers a procedural interface that is often used for rapid prototyping and simple plot generation. Matplotlib is highly extendable and allows for fine-grained control over every aspect of a figure, including lines, fonts, and axes. It can produce publication-quality graphs and charts in numerous formats. In this study, Matplotlib was used to dynamically graph the data collected by the program, which were later used to draw insights about the entire TFSI dataset.

Plotly Express is a high-level Python visualization library that functions as a wrapper for the core Plotly.py library [63]. It provides a simple, concise syntax for creating a wide variety of interactive figures, including scatter plots, bar charts, and 3D graphs. Its primary advantage is the ability to generate complex, multi-faceted visualizations with a single function call, often by operating directly on data formats like Pandas DataFrames. By default, it produces rich, web-enabled figures that support zooming, panning, and hover-over tooltips, facilitating interactive data exploration. In this project, Plotly Express was used to create interactive scatterplots between the S–N–S angle and S–N bond length for all the metals investigated in this study. Readers can find the interactive plots prepared with Plotly Express on the Github page accompanying this project [72].

XlsxWriter is a Python module used for creating new Excel files in the .xlsx format [62]. It is designed to write text, numbers, formulas, and images to multiple worksheets. Unlike libraries that read or modify existing files, XlsxWriter is optimized solely for file creation, making it highly efficient for generating large reports or datasets. It provides an extensive API for applying cell formatting, such as fonts, colors, and number formats. In this work, XlsxWriter was employed to export the data into concise Microsoft Excel (xlsx-format) sheets to then prepare publication-ready infographics using OriginPro.

As previously stated, PyCIFTer has two main components: the CIFParser and the accompanying index.py file, both files built with custom in-house code. PyCIFTer was deliberately designed this way to ensure that this tool could be used by the broader research community without the need for immense computer science expertise. The CIFParser class can be used with all its functions without the need for understanding the internal source code for the class.

CIFParser was implemented using standard parsing practices. Namely, the CIFParser class accepts the path to a file as an input and collapses the entire file into its corresponding lines. CIFParser then goes through each corresponding line looking for “_atom_site_fract_z” to validate that a given CIF-format file contains structural information. In this parsing procedure, the cell values of a given unit cell described in the CIF are also stored in a HashMap for later use in the program or for analysis involving unit cell values.

In response to a reviewer’s suggestion, an investigation was made into whether the results of the analysis of TFSI-containing structures were dependent on temperature. No dependence on temperature was found, but, for clarity, the results of the investigation are given in the Supporting Information on pp. S12–S16.

## 4. Conclusions

A computer program denoted PyCIFTer was developed to facilitate large-scale structural analysis of TFSI-containing species by utilization of experimental atomic coordinate data from X-ray diffraction studies found in the Cambridge Structural Database (CSD). PyCIFTer is capable of rapidly analyzing hundreds of structures in rapid succession and can extract desirable structural parameters for comparisons between classes of species and extrapolation of trends. The approach used in this work for the development of PyCIFTer was designed to avoid tedious one-at-a-time interrogations of structures, a prospect unreasonable in this case considering the large number of TFSI-containing species that have been crystallized in prior work. Taken together, this work demonstrates the usefulness of modular workflows for sequential/batch analysis of structural data from XRD. This study also highlights some of the strategies that are needed from a computer science perspective to achieve analyses of this type and the importance of cross-disciplinary collaboration in facilitating projects in chemical informatics.

## Data Availability

The original contributions presented in this study are included in the article and the associated supplementary materials. PyCIFTer is available online via GitHub [72]. Further inquiries can be directed to the corresponding authors.

## Supplementary Materials

The following supporting information can be downloaded at https://www.mdpi.com/article/10.3390/molecules31010018/s1, PDF-format file containing supplemental figures and data.
