# Supplementary material for: Analysis of Bis(trifluoromethylsulfonyl)imide Interactions with Metal Cations Through a Chemical Informatics Approach
Source: Molecules. 2025 Dec 20;31(1):18. doi: 10.3390/molecules31010018 (PMC12786564; doi:10.3390/molecules31010018)
Supplement: Supplementary file 1 [file molecules-31-00018-s001.zip › molecules-3983885-supplementary.pdf]

Supporting Information for:

Analysis of Bis(trifluoromethylsulfonyl)imide Interactions with Metal Cations Through a Chemical Informatics Approach

*Tej Gumaste,[a,b] Fynn L. Cooper,[a] and James D. Blakemore[a,]**

[a] Department of Chemistry, University of Kansas,
1567 Irving Hill Road, Lawrence, Kansas 66045, USA

[b] Department of Electrical Engineering and Computer Science, University of Kansas,
1520 West 15th Street, Lawrence, Kansas 66045, USA

* To whom correspondence should be addressed: blakemore@ku.edu

Contents

Tau Determination – Sensitivity Analysis S2
Graph Export Examples S3
Analysis of Structural Results S5
Chemical Informatics Results as a Function of Temperature S12

Tau Determination – Sensitivity Analysis

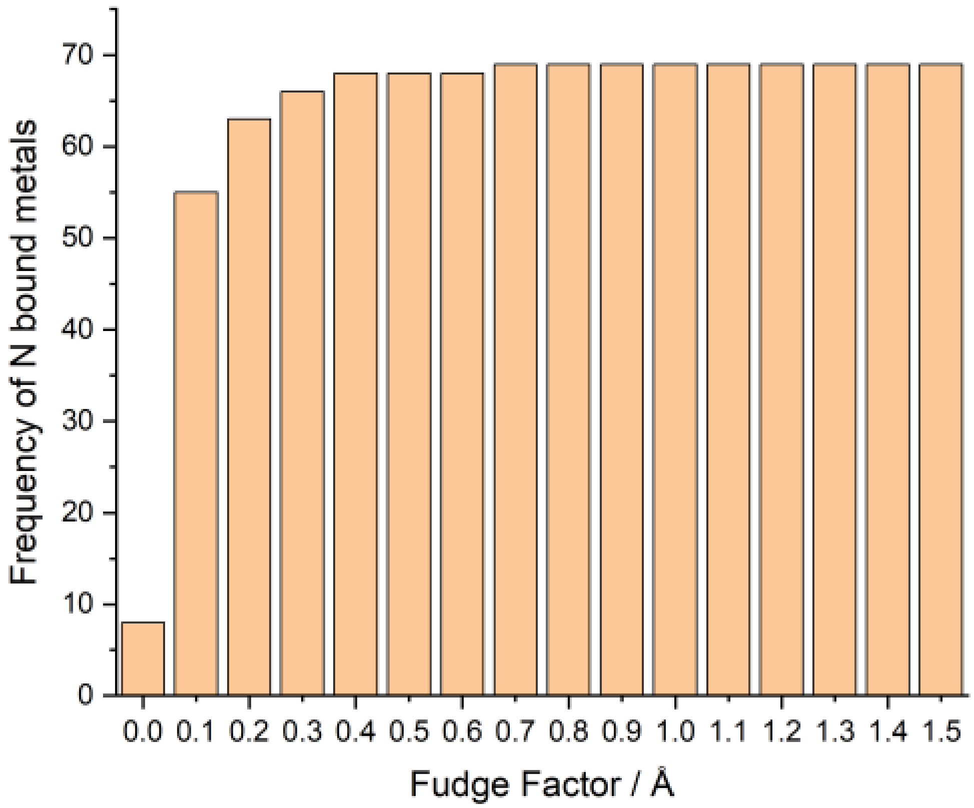

Figure S1. Histogram showing the relationship between the number of structures computed to feature a TFSI that is N-bound to a metal as a function of the τ-value. In our chosen computational procedure, a given metal was considered to be bound to the N-atom of the TFSI core structure if the sum of the covalent radii of the N-atom of TFSI and the relevant metal (M) plus an empirical tolerance "fudge factor" (denoted τ) of 1 Å was greater than the raw distance between N and M. Considering this approach, the value of τ determines the threshold at which M-N bonding appears to become significant. This histogram demonstrates how the τ-value of 1.0 was selected, considering that it allows computational capture of virtually all of the structures which appear to indeed feature TFSI that is N-bound to M.

Graph Export Examples

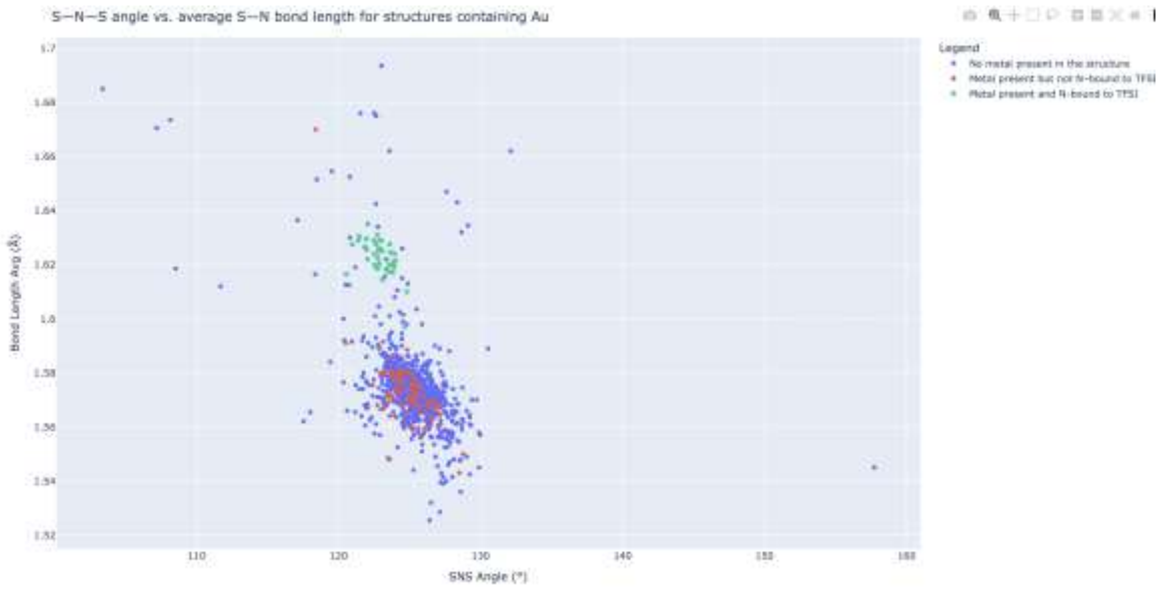

Figure S2. Image of an interactive plot showing the distribution of structures as functions of S–N–S angle and average S–N bond length. In the native plot, moving the cursor over a specific point reveals the properties of the given structure including the values of the S–N–S angle and the S–N bond length as well as the CSD refcode for the corresponding entry in the Cambridge Structural Database. The clustering of structural hits for Au indicates that coordination of Au to the N atom of TFSI results in a significant deviation of the geometric/structural properties of the TFSI core structure.

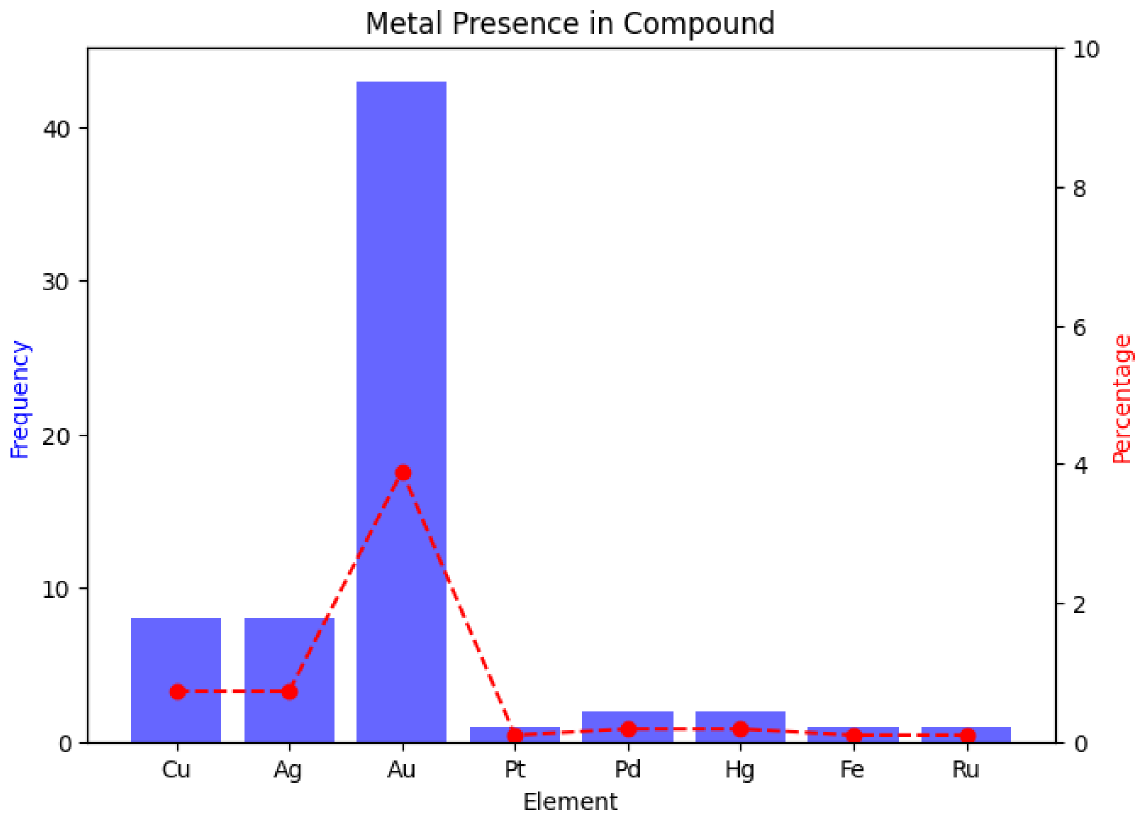

Figure S3. Plot of results from the metal coordination analysis. This figure was prepared using Python, but conveys the same information as in Figure 6 of the main text. As in Figure 6, the left y-axis, associated with the bars shown for each element, denotes the raw frequency of hits of [SNS] moieties coordinated to metals. Each bar corresponds to the individual number of metal-bound moieties with the noted metal/element names. The right y-axis, associated with the red points connected by dashed lines) denotes the percentage of [SNS] moieties coordinated to the individual metals with respect to the entire [SNS] moiety count.

Analysis of Structural Results

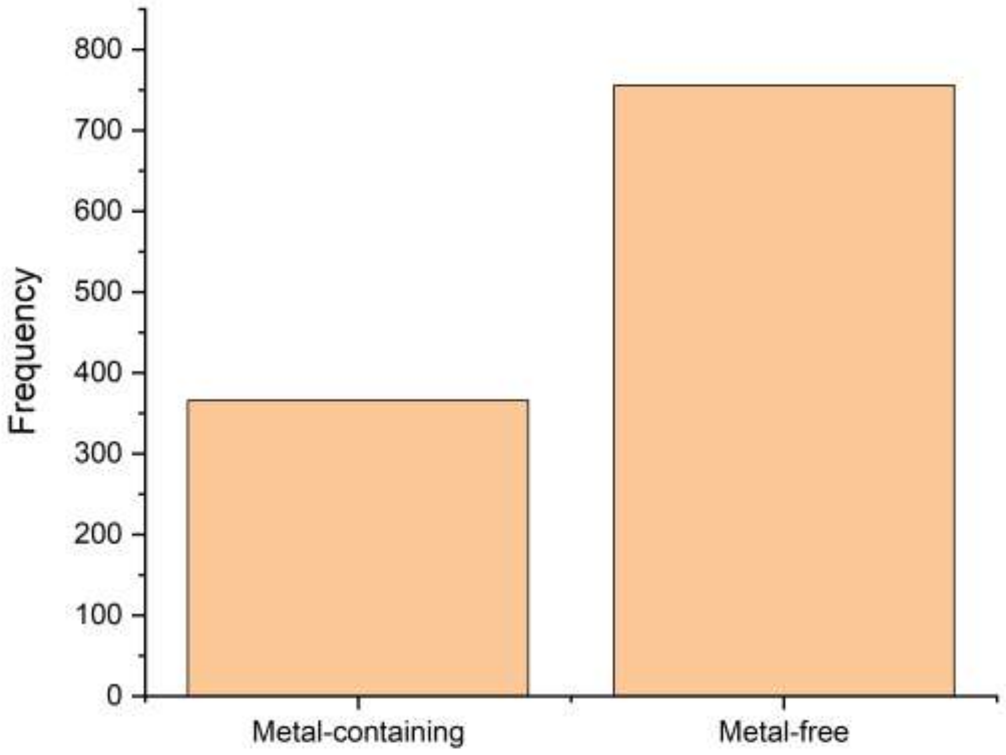

Figure S4. Comparison of the number of metal-containing and metal-free structures. The percentage of the structures containing a metal was found to be 32.3%, while the percentage lacking a metal was 67.7%. The dataset for this comparison was obtained from the Cambridge Structual Database by searching for all structures featuring the TFSI core framework (which has the molecular formula $N(SO_2CF_3)^{2-}$) as specified by connectivity only and agnostic of the individual atom-to-atom bond type.

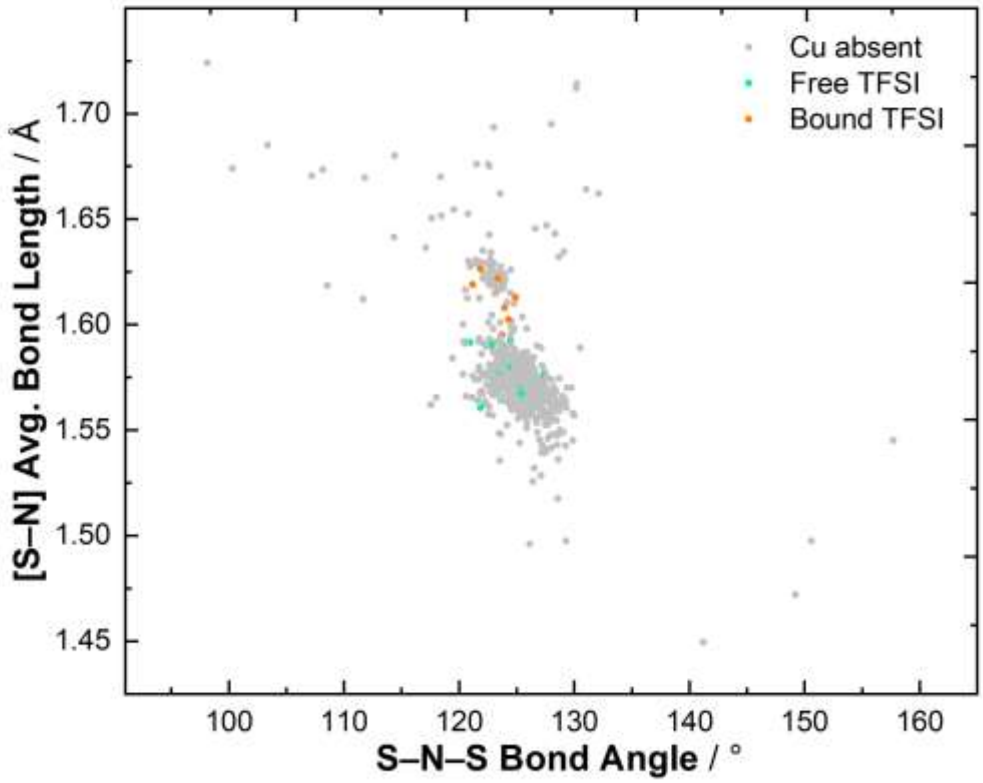

Figure S5: Plot focusing on results for copper cations showing the distribution of structures as functions of S–N–S angle and average S–N bond length. The orange points correspond to [SNS] moieties that are N-bound to copper, the light green points correspond to [SNS] moieties that were found in structures that contain copper atoms (but in which the copper is not N-bound to a TFSI moiety), and the gray points correspond to [SNS] moieties that are found in structures that do not contain copper.

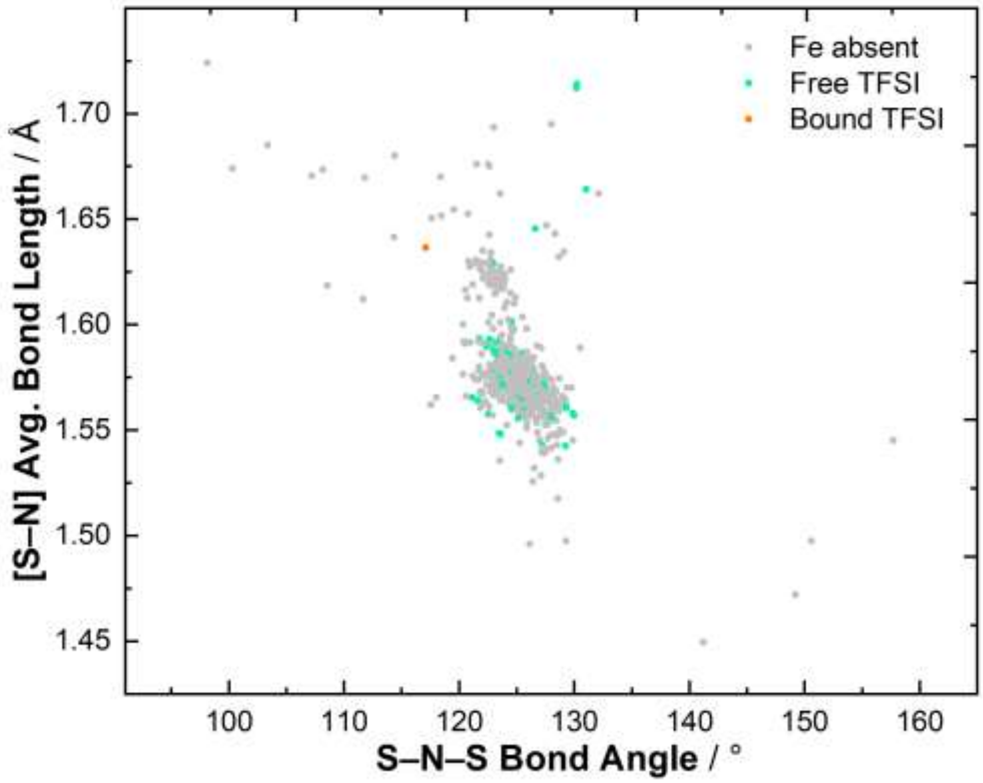

Figure S6: Plot focusing on results for iron cations showing the distribution of structures as functions of S–N–S angle and average S–N bond length. The orange points correspond to [SNS] moieties that are N-bound to iron, the light green points correspond to [SNS] moieties that were found in structures that contain iron atoms (but in which the iron is not N-bound to a TFSI moiety), and the gray points correspond to [SNS] moieties that are found in structures that do not contain iron.

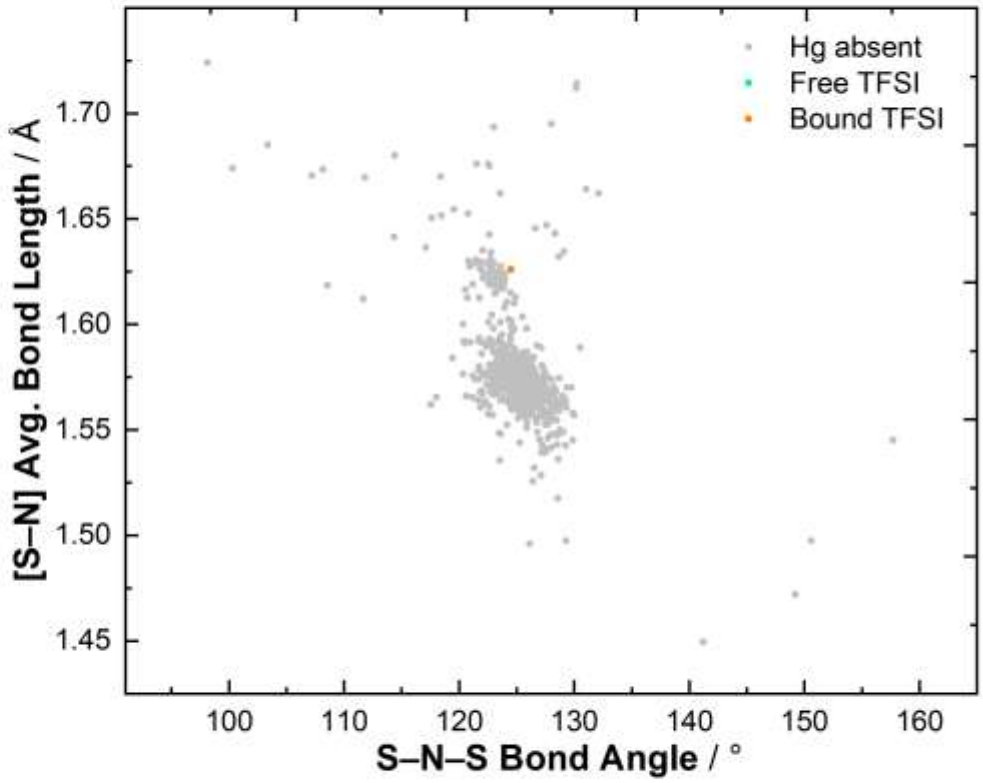

Figure S7: Plot focusing on results for mercury cations showing the distribution of structures as functions of S–N–S angle and average S–N bond length. The orange points correspond to [SNS] moieties that are N-bound to mercury, the light green points correspond to [SNS] moieties that were found in structures that contain mercury atoms (but in which the mercury is not N-bound to a TFSI moiety), and the gray points correspond to [SNS] moieties that are found in structures that do not contain mercury.

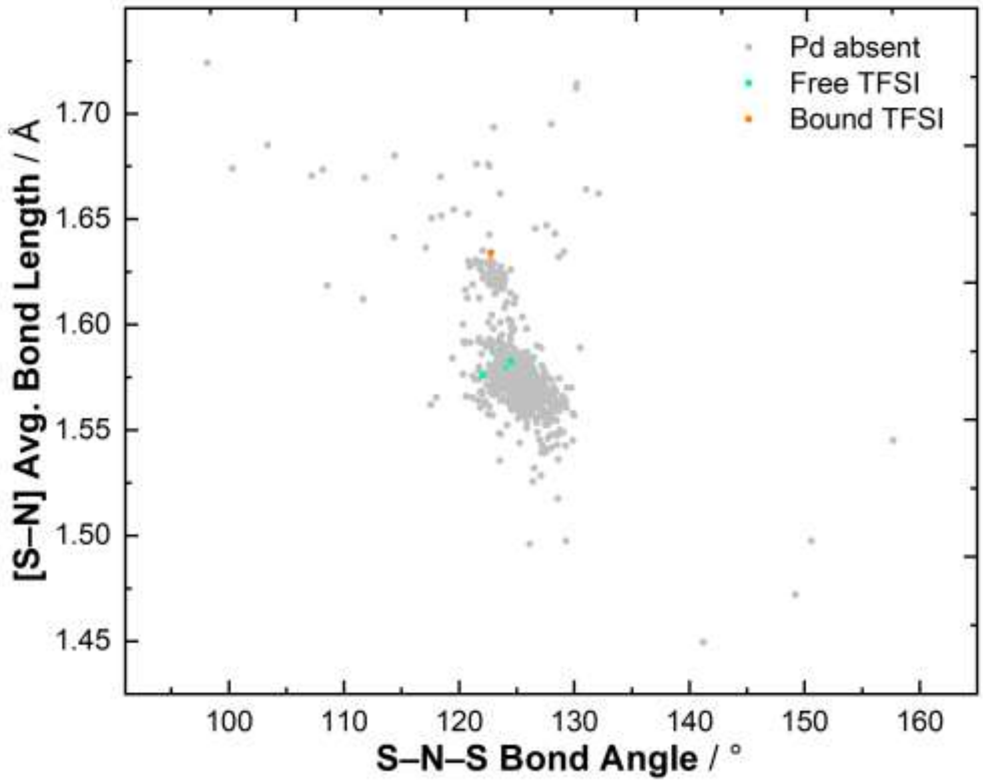

Figure S8: Plot focusing on results for palladium cations showing the distribution of structures as functions of S–N–S angle and average S–N bond length. The orange points correspond to [SNS] moieties that are N-bound to palladium, the light green points correspond to [SNS] moieties that were found in structures that contain palladium atoms (but in which the palladium is not N-bound to a TFSI moiety), and the gray points correspond to [SNS] moieties that are found in structures that do not contain palladium.

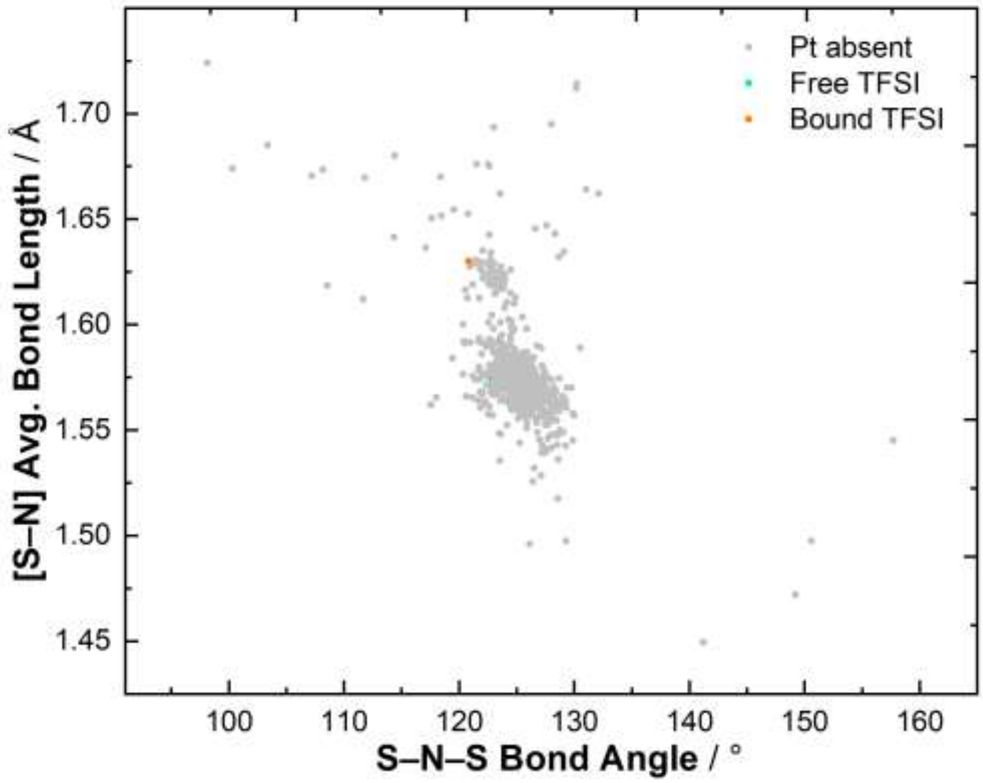

Figure S9: Plot focusing on results for platinum cations showing the distribution of structures as functions of S–N–S angle and average S–N bond length. The orange points correspond to [SNS] moieties that are N-bound to platinum, the light green points correspond to [SNS] moieties that were found in structures that contain platinum atoms (but in which the platinum is not N-bound to a TFSI moiety), and the gray points correspond to [SNS] moieties that are found in structures that do not contain platinum.

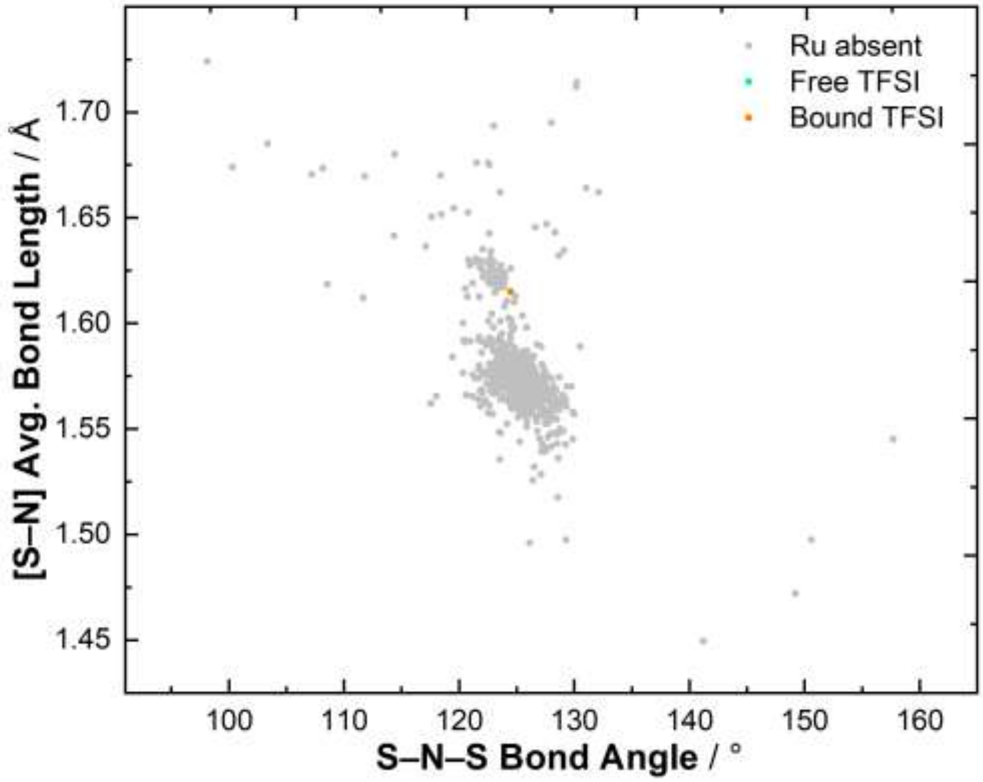

Figure S10: Plot focusing on results for ruthenium cations showing the distribution of structures as functions of S–N–S angle and average S–N bond length. The orange points correspond to [SNS] moieties that are N-bound to ruthenium, the light green points correspond to [SNS] moieties that were found in structures that contain ruthenium atoms (but in which the ruthenium is not N-bound to a TFSI moiety), and the gray points correspond to [SNS] moieties that are found in structures that do not contain ruthenium.

Chemical Informatics Results as a Function of Temperature

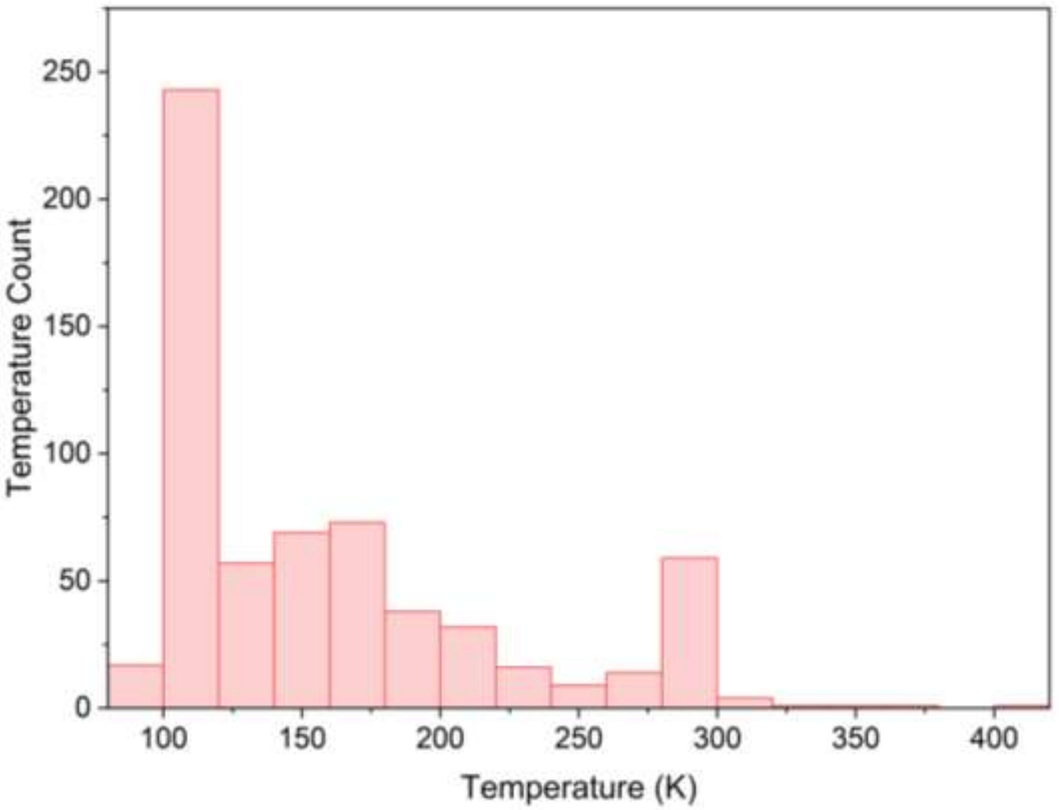

Figure S11: Histogram showing the number of CIF-format files featuring TFSI motifs (in the dataset used for the analysis described in this report) as a function of the temperature used for experimental data collection. The bin size was set at 20 K. As shown in the histogram, most of the data sets were collected at reduced temperatures, presumably with a stream of cold nitrogen gas in each case. The most common data collection temperature is around 100 K. Another common temperature for data collection appears to be near standard/room temperature, ca. 298 K.

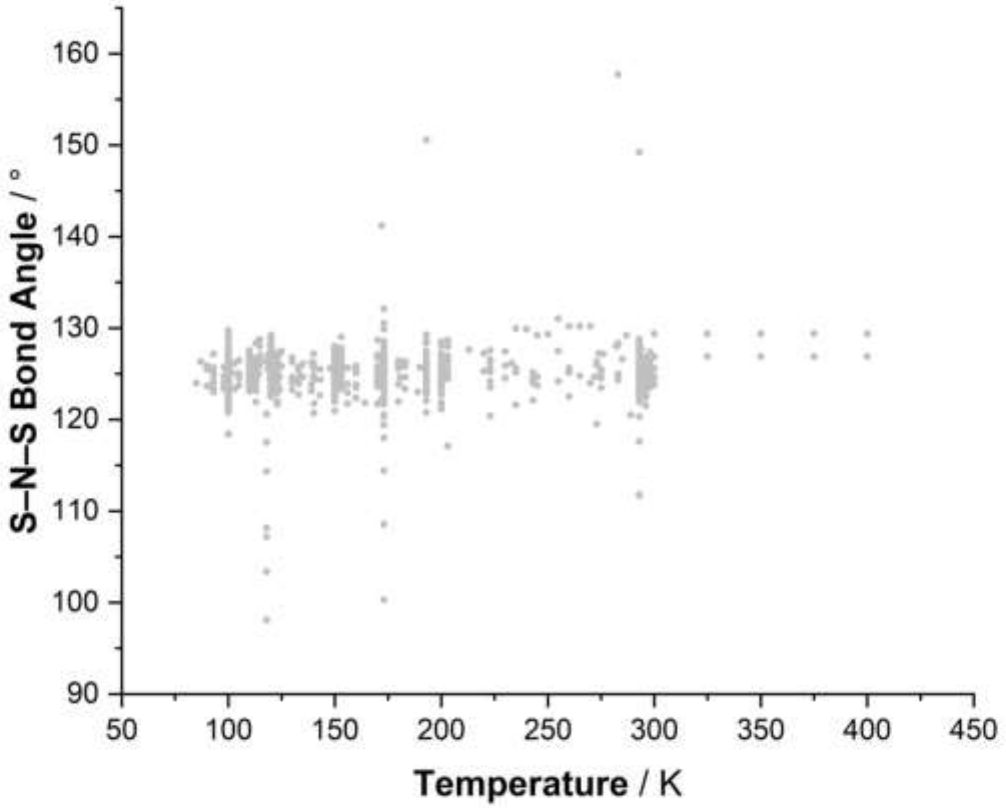

Figure S12: Scatter plot showing that there is no significant dependence of the S–N–S angle of individual TFSI moieties and the temperature of the experimental data collection.

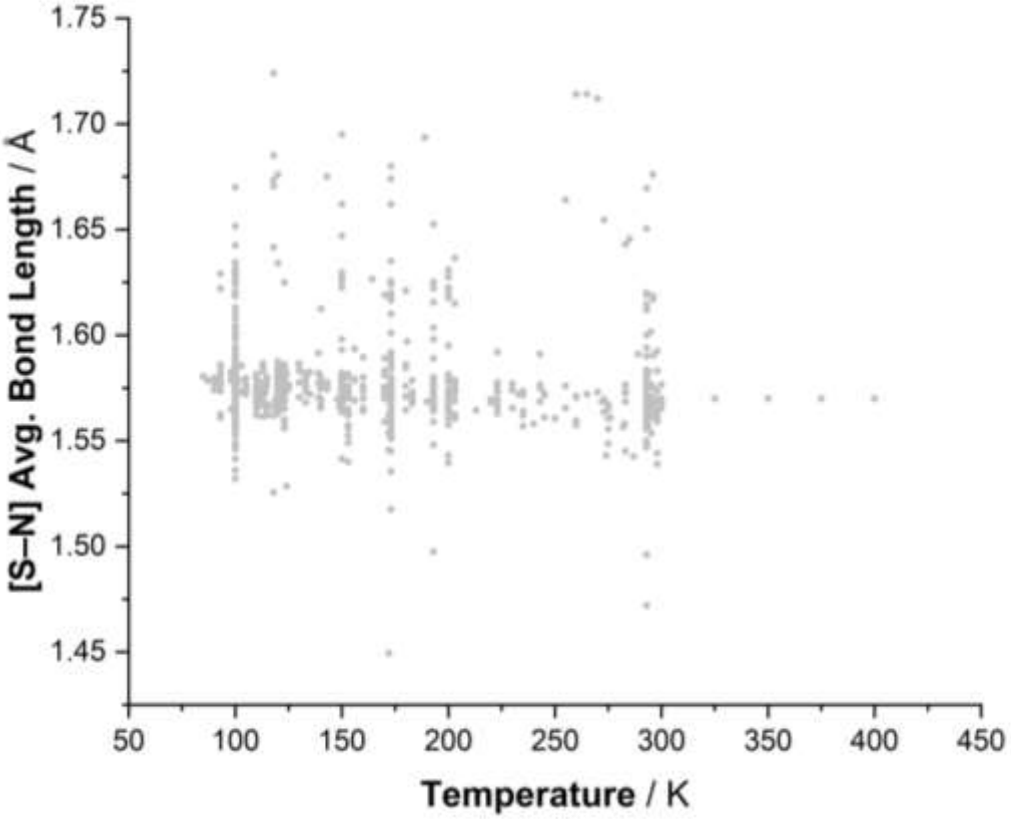

Figure S13: Scatter plot showing that there is no significant dependence of the average S–N bond length of individual TFSI moieties and the temperature of the experimental data collection.

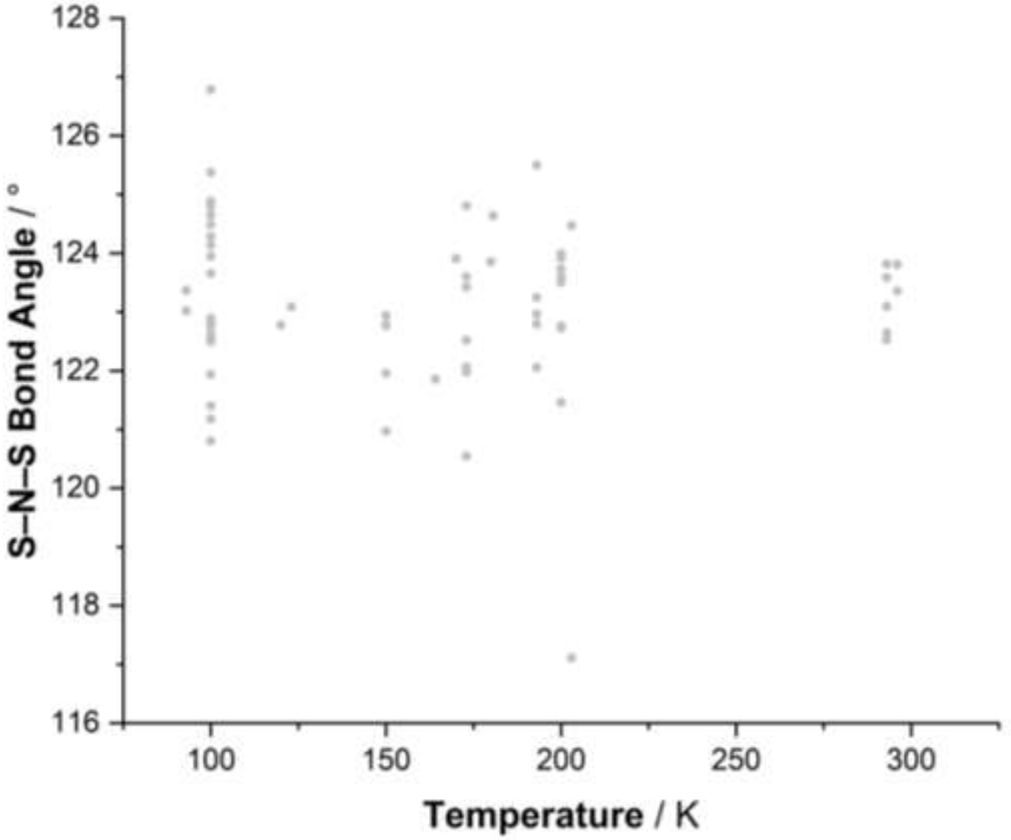

Figure S14: Scatter plot of the S–N–S angle of individual TFSI moieties which are bound to metal cations as a function of the temperature of the experimental data collection. There are significantly fewer data points in this figure compared to Figure S12 because there are many fewer metal-bound TFSI moieties in the full set of CIF-format files used in this work. Although the total range of bond angles is somewhat greater at 100 K compared to ca. 298 K, the range of bond angles measured across all temperatures is similar. Thus, we conclude that there is virtually no effect of the data collection temperature on the structural properties of TFSI.

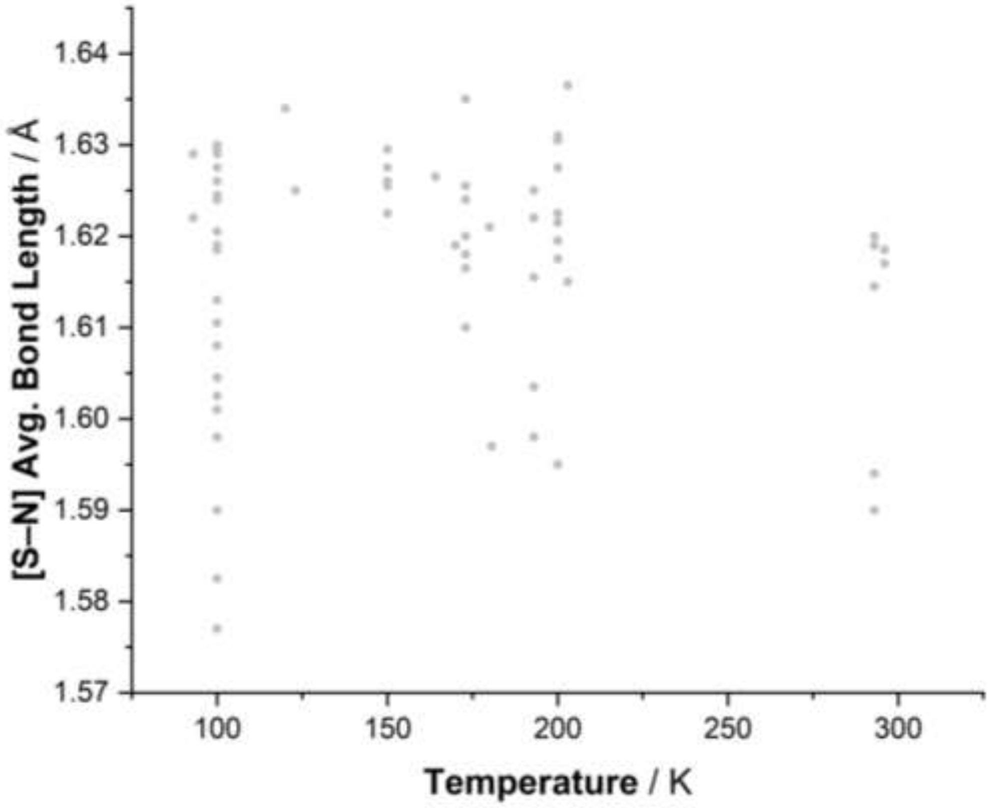

Figure S15: Scatter plot of the S–N bond length of individual TFSI moieties which are bound to metal cations as a function of the temperature of the experimental data collection. There are significantly fewer data points in this figure compared to Figure S13 because there are many fewer metal-bound TFSI moieties in the full set of CIF-format files used in this work. Although the total range of bond lengths is marginally greater at 100 K compared to ca. 298 K, the range of bond lengths measured across all temperatures is similar. Thus, we conclude that there is virtually no effect of the data collection temperature on the structural properties of TFSI.
